# Trehalose-6-phosphate promotes fermentation and glucose repression in *Saccharomyces cerevisiae*

**DOI:** 10.15698/mic2018.10.651

**Published:** 2018-10-01

**Authors:** Rebeca L. Vicente, Lucie Spina, Jose P.L. Gómez, Sebastien Dejean, Jean-Luc Parrou, Jean Marie François

**Affiliations:** 1 LISBP; UMR INSA-CNRS 5504 & INRA 792; Toulouse, France.; 2 Fundación Alfonso Martín Escudero; Madrid, Spain.; 3 Institut de Mathématiques de Toulouse, 118 route de Narbonne, F-31062 Toulouse, France.; 4 Toulouse White Biotechnology Center, UMS INSA-INRA-CNRS, F-31520 Ramonville.

**Keywords:** TPS1, trehalose 6-phosphate, glycolysis, flux sensing, Crabtree effect, glucose repression, Saccharomyces cerevisiae

## Abstract

The yeast trehalose-6-phosphate synthase (Tps1) catalyzes the formation of trehalose-6-phosphate (T6P) in trehalose synthesis. Besides, Tps1 plays a key role in carbon and energy homeostasis in this microbial cell, as shown by the well documented loss of ATP and hyper accumulation of sugar phosphates in response to glucose addition in a mutant defective in this protein. The inability of a *Saccharomyces cerevisiae tps1* mutant to cope with fermentable sugars is still a matter of debate. We reexamined this question through a quantitative analysis of the capability of *TPS1* homologues from different origins to complement phenotypic defects of this mutant. Our results allowed to classify this complementation in three groups. A first group enclosed *TPS1 *of* Klyveromyces lactis *with that of* S. cerevisiae *as their expression in* Sctps1 *cells fully recovered wild type metabolic patterns and fermentation capacity in response to glucose. At the opposite was the group with *TPS1* homologues from the bacteria *Escherichia coli* and *Ralstonia solanacearum*, the plant *Arabidopsis thaliana* and the insect *Drosophila melanogaster* whose metabolic profiles were comparable to those of a *tps1* mutant, notably with almost no accumulation of T6P, strong impairment of ATP recovery and potent reduction of fermentation capacity, albeit these homologous genes were able to rescue growth of Sc*tps1* on glucose. In between was a group consisting of *TPS1* homologues from other yeast species and filamentous fungi characterized by 5 to 10 times lower accumulation of T6P, a weaker recovery of ATP and a 3-times lower fermentation capacity than wild type. Finally, we found that glucose repression of gluconeogenic genes was strongly dependent on T6P. Altogether, our results suggest that the TPS protein is indispensable for growth on fermentable sugars, and points to a critical role of T6P as a sensing molecule that promotes sugar fermentation and glucose repression.

## INTRODUCTION

The yeast *Saccharomyces cerevisiae* is widely used as a eukaryotic model organism for fundamental research. In addition, it is the most utilized microorganism in traditional biotechnological applications such as bread-making and fermented drinks 1, and deserves a great desirability towards the exploitation of renewable carbon sources for fuels and commodity chemicals 2,3,4,5. These applications often involve rational design of the carbon metabolic pathways to increase performance and robustness, quantitatively expressed as production titer, yield and productivity. This metabolic rewiring requires at first glance an extremely well detailed knowledge of the metabolic networks and their regulation.

Glycolysis is the backbone of the carbon metabolism in *S. cerevisiae *and even after more than 50 years of intensive research 6, the regulation of this pathway is not completely understood. A still unsolved issue concerns the role of the trehalose-6-phosphate synthase (TPS) complex in carbon and energy metabolism of the yeast *S. cerevisiae. *This complex contains four subunits, namely Tps1, Tps2, Tps3 and Tsl1. The subunit encoded by *TPS1 *catalyzes the formation of trehalose-6-phosphate (T6P) from glucose-6-phosphate (G6P) and UDP-glucose. T6P is then dephosphorylated by the subunit encoded by *TPS2*. Subunits encoded by *TPS3 *and *TSL1 *do not harbor any known catalytic function but may participate in the integrity of the TPS complex 7,8. The deletion of *TPS1*, not only prevents the synthesis of trehalose, an α-D-glucopyranosyl-(1-> 1)-a-D-glucopyranoside, that serves both as storage carbohydrate and stress protectant in yeast 7,9, but most importantly, it leads to a growth defect on fermentable sugars 8,10. This defect is witnessed by a massive accumulation of sugar phosphates and depletion of ATP upon addition of rapidly assimilated sugars to nutrient-starved yeast or yeast cultivated on non-fermentative carbon sources 11,12. Two models have been proposed to account for this glycolytic defect phenotype, namely an over-activity of the major yeast hexokinase encoded by *HXK2 *due to the loss of its inhibition by T6P 13, and a depletion of intracellular Pi due to hyper-accumulation of sugar phosphates leading to an inactive glyceraldehyde-3-P dehydrogenase 11. More recently, van Heerden *et al *14 revisited these two models through the combination of flux analysis using 13C labelling experiments and mathematical modelling. They concluded about the importance of Pi-dynamic in the startup of glycolysis, which can be brought about by an ATP hydrolysis, resulting from trehalose metabolic cycling. Nonetheless, this last model does not consider that a mutant defective in *TPS2 *does not exhibit any apparent glycolytic defects, except a very high accumulation of T6P 15. In addition, besides this glycolytic defect, a yeast mutant defective in *TPS1 *exhibits other multiple and apparently unrelated physiological defects, such as hyper-accumulation of glycogen 16, sporulation deficiency 17, higher uncoupling of the respiratory chain 18, reduced phospholipid production 19, lack of gluconeogenesis repression 20, as well as a high sensitivity to temperature and oxidative stress 21. Altogether, these results underline a wider role of Tps1 in the regulation of *S. cerevisiae *metabolism.

To further document on the importance of the Tps1 protein and its product T6P in yeast sugar metabolism, we decided to revisit previous works dealing with complementation studies of a *S. cerevisiae tps1*Δ mutant (*Sctps1*Δ) with *TPS1 *homologues from different origins, including bacteria, fungi, plants and insects. This work was motivated by the fact that these complementation studies were merely qualitative, employing episomic plasmids in which *TPS1 *homologues were cloned either under their own promoter or under *ADH1 *or *PGK1 *promoter. Also, these complementation assays were carried out with *tps1 *mutants from different strain backgrounds 22,23,24,25,26,27, which hampered any comparison between the data. In addition, a recent result of our group showed that *S. cerevisiae tps1*Δ mutant recovered growth on glucose after transformation with the *Yarrowia lipolytica TPS1 *(*YlTPS1*) cloned in an episomic plasmid under the *PGK1 *promoter, even though T6P was barely detectable 15. Conversely, Bonini *et al*. 25 reported a partial glucose growth recovery of a *Sctps1*Δ upon expression of the *E. coli otsA *cloned in an episomic plasmid under *PGK1 *promoter while levels of T6P were almost comparable to those in wild type yeast. Therefore, in order to seek further on the role of Tps1 and T6P in yeast sugar fermentation, we compared the potency of various *TPS1 *homologues to quantitatively complement phenotypic traits of a *tps1*Δ mutant made in the physiologically well characterized CEN.PK strain 28. This comparative study was carried out by expressing these homologous genes on the same centromeric vector under a constitutive *PGK1 *promoter, instead of the *TPS1 *promoter, to avoid unpredicted transcriptional control of this gene. This work led us to propose that the Tps1 protein is indispensable for growth on a fermentable carbon source, whereas T6P may act as a sensing molecule promoting fermentation and glucose repression.

## RESULTS

### Rationale to select *TPS1* homologues to complement the phenotype of the Sc*tps1*Δ mutant

**Figure 1:**
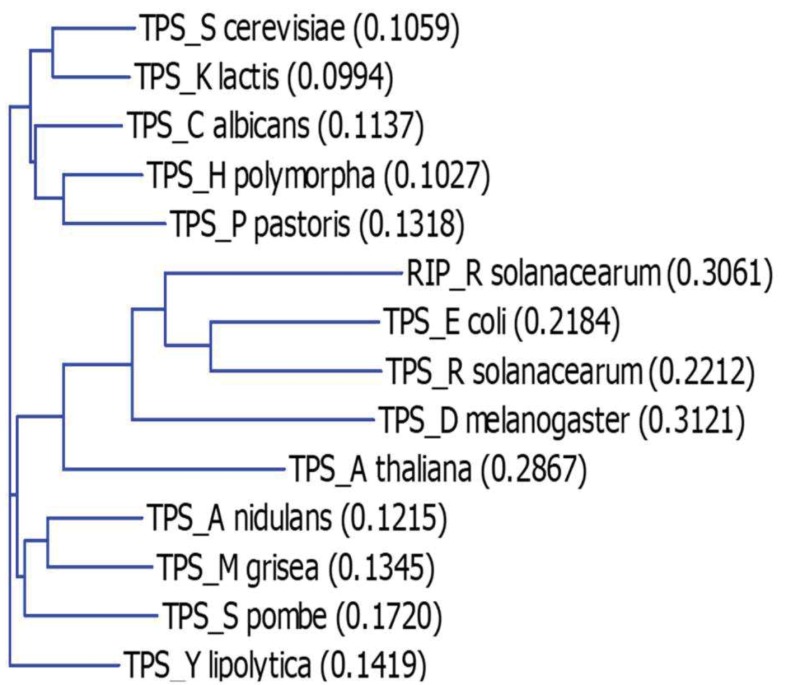
FIGURE 1: Phylogenetic tree of TPS protein homologues. Phylogenetic tree calculation is based on a sequence distance method and utilizes the Neighbour Joining algorithm of Saitou and Nei 31.

Based on a phylogenetic analysis of the TPS proteins reported earlier by Avonce *et al *29, we selected *TPS1 *genes that belong to the main branches of the tree topologies, and combined this selection with previous works reporting complementation of the *Sctps1*Δ mutant by the selected genes. Accordingly, we performed a new phylogenetic analysis with the retained 14 TPS proteins. This analysis grouped these proteins into 3 subgroups (Fig. 1). In the first group are TPS proteins from the yeast species including *S. cerevisiae, K. lactis, Candida albicans, Hansenula polymorpha *and *Pichia pastoris*. A second subgroup comprises TPS from the yeast *Schizosaccharomyces pombe *and the two filamentous fungi *Aspergillus nidulans *and *Magnaporthe grisea*. The position of TPS from *Yarrowia lipolytica *is somehow in forefront of these two subgroups. The TPS protein from the bacteria *E. coli *and *R. solanacearum *composed the third distinct subgroup, which also contains the protein from the insect *D. melanogaster*. The TPS protein from the plant *A. thaliana *encoded by *TPS1 *linked in between the second and the third subgroup. Comparison of amino acids sequence alignment of these TPS proteins confirmed that they all harbored the residues involved in the binding of the substrates G6P and UDPGlc (see Fig. S1). This alignment also revealed an N-terminal extension of the *A. thaliana *TPS protein which is also present in the Rip protein of *R. solanacearum *(Fig. S2). It was shown that this N-terminal extension has an inhibitory effect on the catalytic activity of the TPS protein 30. This alignment also underscores an important C-terminal extension of more than 300 amino acids length for the TPS protein of *A. thaliana*, *P. pastoris *and *D. melanogaster*.

### Striking differences in the potency of *TPS1 *homologues from different origins to recover growth of *Sctps1*Δ on a fermentable sugar 

**Figure 2:**
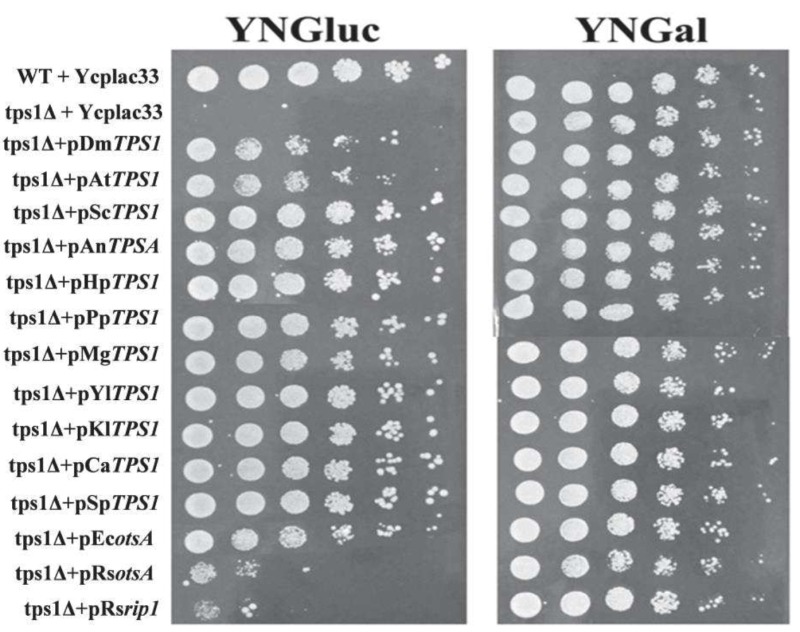
FIGURE 2: Growth assay of *S. cerevisiae tps1*Δ mutant transformed with *TPS1 *from different organisms on solid media. The *Sctps1*Δ strain transformed with YcpLac33 plasmid bearing *TPS1 *homologue was cultivated on YN trehalose 1 % till 5 OD_600_ units. 10 μl of serial dilution from this preculture was spotted on YN agar plate containing glucose or galactose at 2% (w/v). Growth was scored after 2 days at 30°C.

To investigate the ability of *TPS1 *homologues from bacteria, fungi, plants and insects to complement phenotypes of *S. cerevisiae tps1*Δ strain (Sc*tps1*Δ), we cloned their CDS (coding sequence) in the centromeric YCplac33 plasmid under the Sc*PGK1 *promoter. As shown in Fig. 2, all the *TPS1 *homologous genes could complement the glucose-negative phenotype of a Sc*tps1*Δ mutant on solid media although with different efficiency. Indeed, the potency for growth recovery was lower with the plant, insect and *E. coli TPS1 *homologous genes than with yeast and filamentous fungi counterparts, and was only perceptible with the two *R. solanacearum TPS1 *homologous genes, namely *otsA *and *rip1*. The weak capacity of these *TPS1 *homologues to complement the growth defect of Sc*tps1*Δ could not be ascribed to a lack of gene expression, since transcripts of these genes were between 1 to 2,5-fold higher than that of the endogenous *TPS1 *in the wild type as determined by RT-qPCR (Fig. S3). It could be noticed that the transcription of *TPS1 *homologues from *E. coli, A. thaliana *and *D. melanogaster *was among the highest, in spite of a weaker capacity of these genes to complement the glucose growth defect of a *Sctps1*Δ.

**Figure 3:**
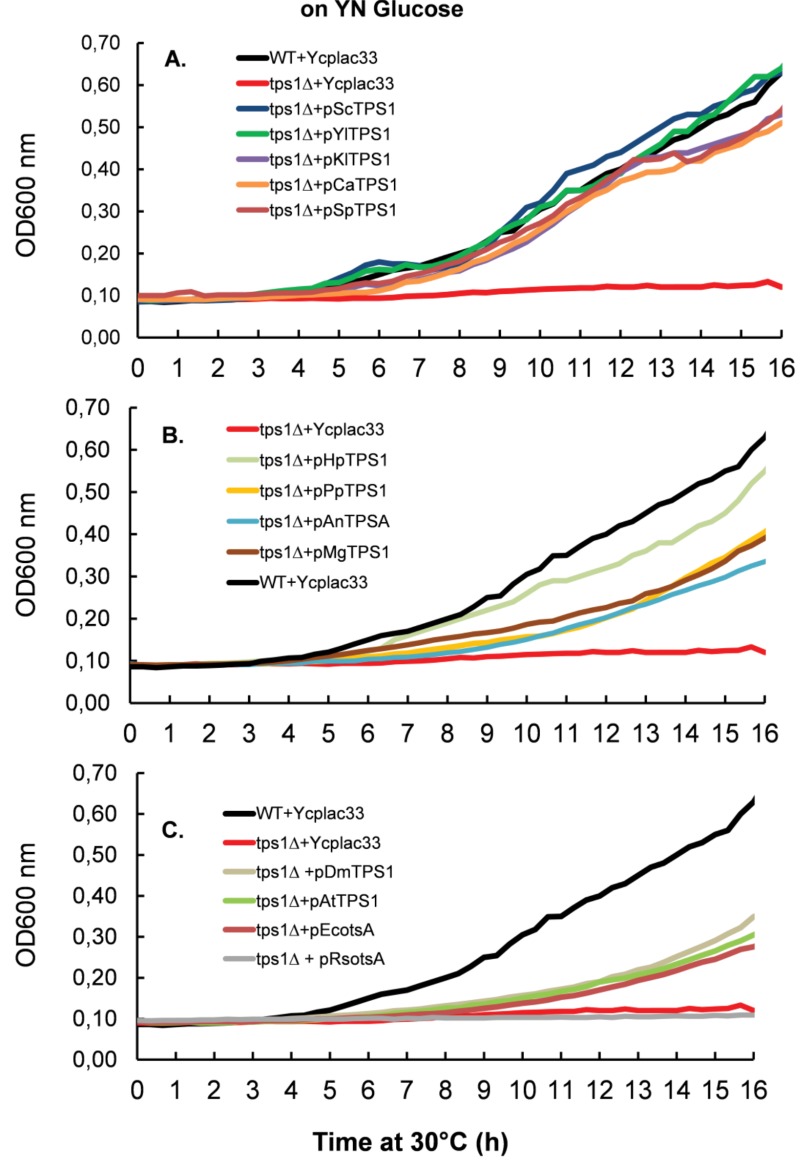
FIGURE 3: Resumption of growth of *S. cerevisiae tps1* mutant transformed with *TPS1* from different organism on YN glucose media in microtiter plate. **(A)** Complementation of sctps1 with *TPS1* homologs from yeast species. **(B)** Complementation of Sctps1 with *TPSD1* homologs from *P. pastoris*, *H. polymorpha* and the filamentous fungi *A. niger* and *M. grisea*. **(C)** Complementation of sctps1 with *TPS1* homologs from bacteria, plants and drosophila. The experimental design was as in Figure 2 except that the yeast cells were inoculated in 250 µL of YN containing glucose at 2% (w/v). Results shown are the mean of two independent experiments. For the sake of clarity, standard deviation bars have been withdrawn, but overall the SD was in the range of 10 - 15 %.

**Figure 4:**
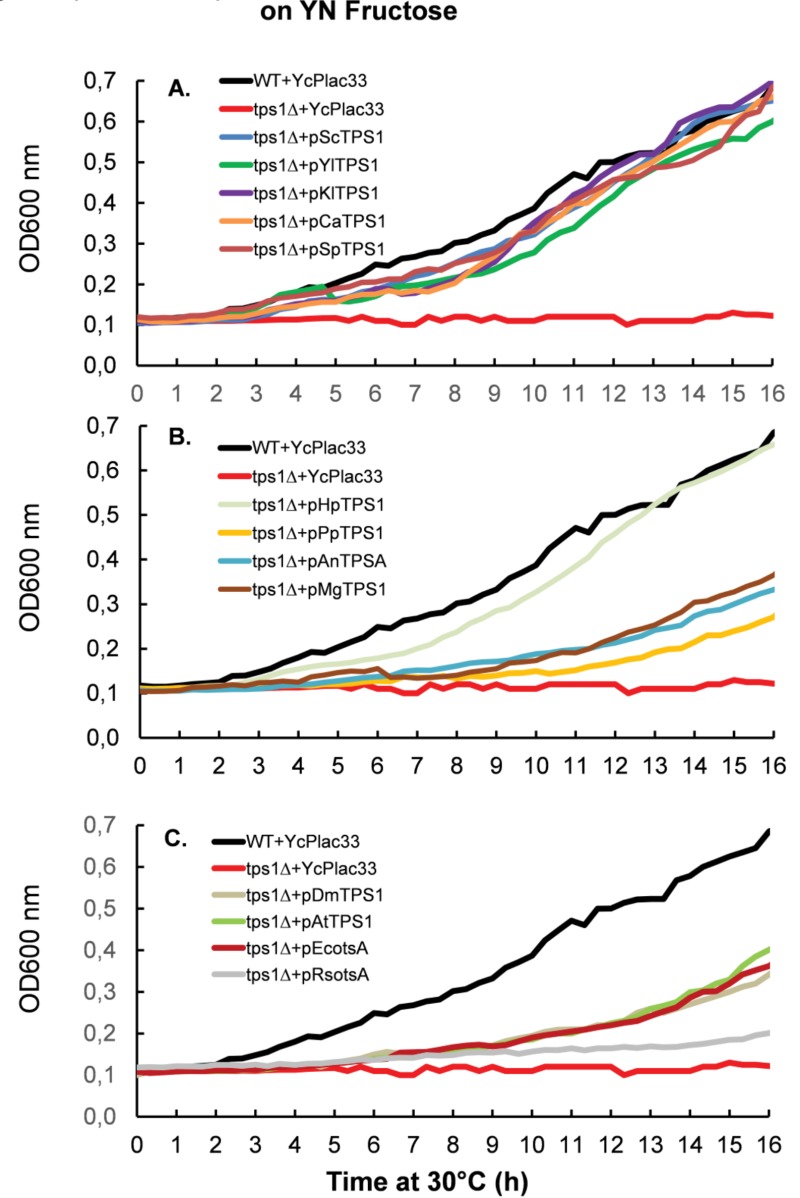
FIGURE 4: Resumption of growth of *S. cerevisiae tps1* mutant transformed with *TPS1* from different organism on YN fructose media in microtiter plate. **(A)** Complementation of sctps1 with *TPS1* homologs from yeast species. **(B)** Complementation of Sctps1 with *TPSD1* homologs from *P. pastoris*, *H. polymorpha* and the filamentous fungi *A. niger* and *M. grisea*. **(C)** Complementation of sctps1 with *TPS1* homologs from bacteria, plants and drosophila. The experimental design was as in Fig.2 except that the yeast cells were inoculated in 250 µL of YN containing fructose at 2% (w/v). For the sake of clarity, standard deviation bars have been withdrawn, but overall the SD was in the range of 10 -15 %.

We next measured the growth rate of Sc*tps1*Δ transformed with the different *TPS1 *homologues on different carbon sources. To this end, all yeast transformants were pre-cultured in the permissive YN medium containing 1% trehalose. As reported earlier, this culture condition mimicked a steady-state glucose limited continuous culture at low dilution rate which favors full respiration 32,33 and can be quickly switched into a respiro-fermentative mode by addition of excess glucose 15. When the cultures reached around 5 units OD_600_, they were diluted 50-fold in 0.35 ml of YN medium containing glucose (Fig. 3) or fructose (Fig. 4). This experiment clearly showed that *TPS1 *homologues do not equally restore growth of the *Sctps1*Δ mutant on these fermentable sugars. With the exception of the *P. pastoris TPS1, *the *TPS1 *homologues of the yeast species were as efficient as the *S. cerevisiae *gene to reestablish growth rate of the Sc*tps1*Δ mutant on these sugars. In contrast, the resumption of growth was retarded in *tps1*Δ expressing *TPS1 *homologues from the filamentous fungi *A. nidulans *and *M. grisea. *This delay in growth resumption was even more exacerbated upon expression of the *E. coli*, *A. thaliana *or *D. melanogaster TPS1 *homologue, with the extreme situation of *Sctps1*Δ expressing *R. solanacearum otsA *that only started to grow after 15 h. Likewise, expression of *R*s*rip1*, the other *TPS1 *homologue of this bacterium 34, as well as the *tps1-156 *allele, encoding for a catalytically dead Tps1 35, failed to recover growth of *Sctps1*Δ after two days of cultivation in glucose or fructose containing YN medium (data not shown). Notwithstanding, the difference in the capacity to resume growth on fermentable sugars, we verified whether *Sctps1*Δ transformed with the different *TPS1 *homologous genes exhibited also difference in their maximal growth rate (μ_max_). As reported in Table 1, we found similar μ_max_ on glucose and fructose as wild type of *Sctps1*Δ complemented with yeast and filamentous fungi *TPS1*, with the exception of the *P. pastoris *and *M. grisea *gene for which the growth rate was 25-35% lower than that of wild type. Also, the maximal growth rate was more than two fold lower in Sc*tps1*Δ transformed with the *TPS1 *homologue from *E. coli*, *A. thaliana *and *D. melanogaster*. A generation time of > 10 h was estimated for the Sc*tps1*Δ expressing *RsotsA*, whereas there was no significant growth of the Sc*tps1*Δ mutant bearing Rs*rip1 *on glucose or fructose. On the other hand, the growth resumption and maximal growth rate of *Sctps1*Δ on the permissive carbon source galactose was not impacted by the expression of any of the different *TPS1 *homologues (Table 1 and supplementary data Fig. S4A). As a conclusion, these data indicated that the presence of the Tps1 protein is needed for growth on rapidly assimilated, fermentable sugars, and additionally suggest that the promptness at which the growth can restart on these sugars very likely requires a catalytic active Tps1 protein.

**Table 1:** Growth rate of *S.cerevisiae* tps1 mutant transformed with TPS1 from various origin in microplate on different carbon sources.

	**Strain name**	**YN glucose**	**YN fructose**	**YN galactose**
	**WT+YcPlac33**	0.29 ± 0.02	0.29 ± 0.03	0.24 ± 0.03
	***tps*1Δ+YCplac33**	NG	NG	0.22 ± 0.03
	***tps*1Δ+pSc*TPS1***	0.29 ± 0.02	0.28 ± 0.03	0.26 ± 0.03
	***tps*1Δ+pSp*TPS1***	0.28 ± 0.02	0.29 ± 0.03	0.25 ± 0.03
	***tps*1Δ+pYl*TPS1***	0.28 ± 0.02	0.29 ± 0.03	0.26 ± 0.03
	***tps*1Δ+pKl*TPS1***	0.29 ± 0.02	0.27 ± 0.03	0.25 ± 0.03
	***tps*1Δ+pCa*TPS1***	0.26 ± 0.02	0.25 ± 0.03	0.23 ± 0.03
	***tps*1Δ+pPp*TPS1***	0.19 ± 0.02	0.15 ± 0.03	0.23 ± 0.03
	***tps*1Δ+pHp*TPS1***	0.27 ± 0.02	0.29 ± 0.03	0.24 ± 0.03
	***tps*1Δ+pAn*TPS1***	0.26 ± 0.02	0.26 ± 0.03	0.24 ± 0.03
	***tps*1Δ+pMg*TPS1***	0.21 ± 0.02	0.22 ± 0.03	0.25 ± 0.03
	***tps*1Δ+pEc*otsA***	0.14 ± 0.02	0.12 ± 0.03	0.24 ± 0.03
	***tps*1Δ+pRs*otsA***	0.07 ± 0.02	0.08 ± 0.03	0.22 ± 0.03
	***tps*1Δ+pRs*rip1***	0.05	0.05	0.21 ± 0.03
	***tps*1Δ+ pDm*TPS1***	0.13 ± 0.02	0.12 ± 0.03	0.24 ± 0.03
	***tps*1Δ+pAt*TPSA***	0.11 ± 0.02	0.13± 0.03	0.24 ± 0.03

The yeast strains were cultivated in microtiter plates containing 350 µl of culture media as described in Material and Methods. The microtiter plates were placed in a thermostated Elisa reader (Biotek) set at 30°C under smooth shaking and read every 15 min. NG: no growth. The values are the mean ± standard deviation of three independent biological experiments.

### Tps1 activity in *Sctps1*Δ transformed with *TPS1* homologues and correlation with trehalose levels

The disparity in the growth behavior of the Sc*tps1*Δ mutant complemented with *TPS1 *homologues of different origins while the expression of these genes was apparently correct (see Fig. S3), prompted us to verify whether this phenotypic complementation was accompanied by a measurable Tps1 activity, which would argue that the TPS proteins from these different origins are active *in vivo *in yeast (Table 2). A first indication that our strategy was valuable was given by the finding that *Sctps1*Δ transformed with the Sc*TPS1 *gene expressed from a CEN plasmid under the *PGK1 *promoter regained the activity of Tps1 and levels of trehalose exactly as the wild type (Table 2). We also found that the Tps1 activity and trehalose levels in *Sctps1*Δ transformed with *K. lactis*, *H. polymorpha*, *A. nidulans *and *C. albicans *could be grouped together with that of wild type and *Sctps1*Δ + pSc*TPS1*. With the exception of the An*TPS1*, this clustering matched the subgroup 1 of our phylogenetic analysis (Fig. 1). At the opposite, a second group which corresponded to cluster 2 in Fig. 1 was composed of *Sctps1*Δ transformed with the TPS protein from the bacteria *E. coli *and *R. solanacearum*, the insect *D. melanogaster *and the plant *A. thaliana*. The activity of Tps1 measured in these transformants was overall ten times lower than that of the wild type. The third group, that also corroborated with the phylogenetic analysis, included *TPS1 *from* S. pombe*, *Y. lipolytica*, and *M. grisea, *and showed a Tps1 activity that was around five times lower than that of wild type but sufficiently high to accumulate high levels of trehalose. Finally, the Tps1 activity in Sc*tps1*Δ expressing *P. pastoris TPS1 *was in the same range as that of the bacterial homologous gene (Table 2). When the trehalose content determined in the different strains was plotted as a function of Tps1 activities, a slightly sigmoidal curve was obtained (see supplementary Fig. S5A), allowing to evaluate that optimal synthesis of this disaccharide activity is obtained with an activity of Tps1 in the range of 30 nmol.min^-1^. mg protein^-1^.

**Table 2:** Activity of Trehalose 6-P synthase (Tps1) and levels of trehalose in the *S. cerevisiae tps1* mutant transformed by TPS1 of various origins.

**Strain ID**	**Tps1 activity nmol/(min.mg protein)**	**Trehalose level (mg/g DCW)**
**WT+YCplac33**	76.7 ± 6.6	106 ± 13.5
**tps1Δ+pSc*TPS1***	61.4 ± 3.30	93 ± 15
**tps1Δ+pKl*TPS1***	48.7± 15.8	97 ± 9
**tps1Δ+pCa*TPS1***	25.3 ± 3.3	86 ± 25
**tps1Δ+pHp*TPS1***	44.6 ± 4.8	50 ± 14
**tps1Δ+pAn*TPS1***	39.1 ± 16.2	87 ± 28
**tps1Δ+pSp*TPS1***	15.7 ± 8.6	75 ± 12
**tps1Δ+pMg*TPS1***	18.6 ± 8.6	45 ± 13
**tps1Δ+pYl*TPS1***	8.3 ± 3.1	64 ± 10
**tps1Δ+pPp*TPS1***	5.9 ± 1.8	36 ± 9
**tps1Δ+pEc*otsA***	5.40 ± 3.8	55 ± 10
**tps1Δ+pRs*otsA***	5.90 ± 2.4	1.1 ± 0.15
**tps1Δ+pRs*rip1***	3.20 ± 1.5	7 ± 1.5
**tps1Δ+pDm*TPS1***	6.6 ± 2.30	15 ± 11
**tps1Δ+pAt*TPS1***	4.0 ± 2.9	1.5 ± 0.5
**tps1Δ+YCplac33**	0.10	0.00

The activity of T6P synthase was measured at 42°C as described in Material and Methods. Results are the mean ± SD of three independent assays. Trehalose was determined in stationary phase approximatively 12 h after glucose exhaustion from the medium, at a time when trehalose content is about to reach its maximum and stabilizes for several hours before consumption 36.

### *Sctps1*Δ complemented with different TPS1 exhibited different metabolite patterns in response to a glucose pulse 

**Figure 5:**
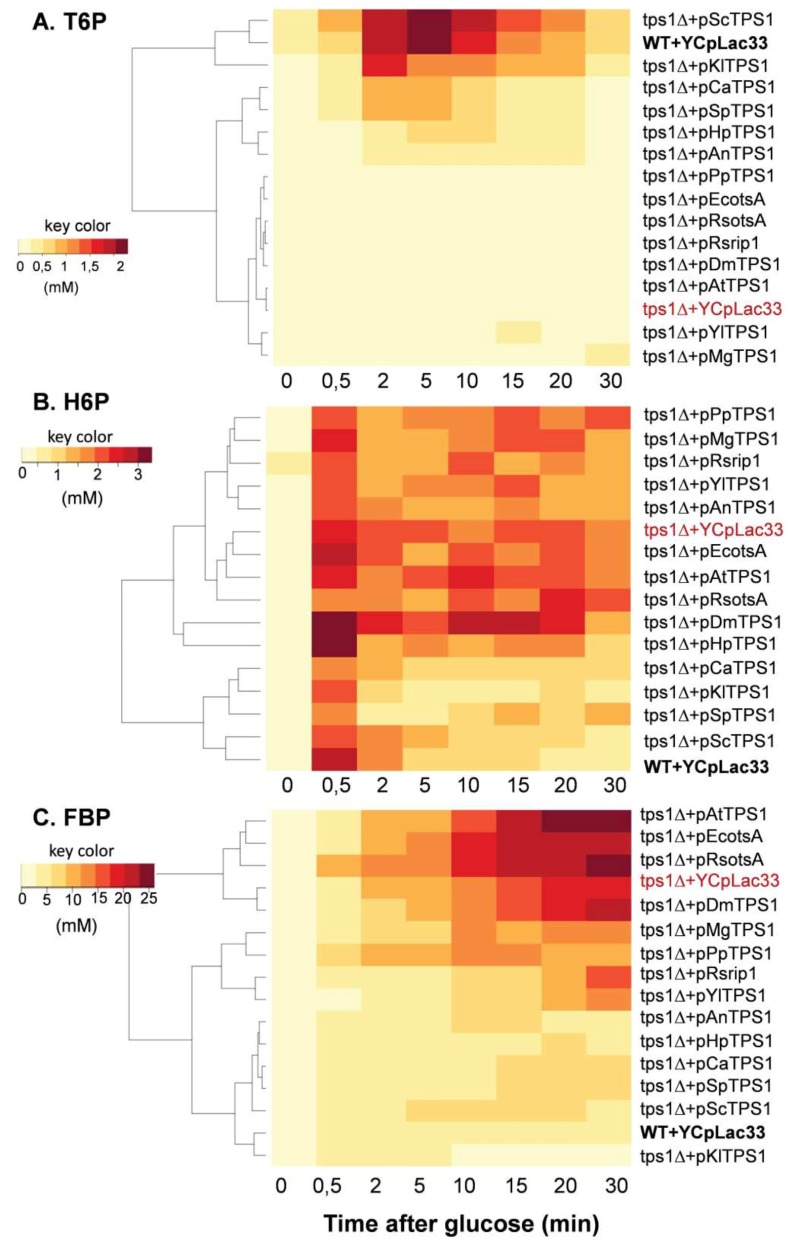
FIGURE 5: Heat map representation of the change in T6P (A), H6P (B) and FBP (C) concentration in *S. cerevisiae tps1 *mutant expressing* TPS1 *from different organisms in response to a glucose pulse. The *Sctps1* strain transformed with YCpL33 plasmid bearing *TPS1* homologue was cultivated on YN Trehalose 1% till 5 OD_600_ units. Then, glucose (10 g/L final concentration) was added and samples (5 ml) at different times were rapidly collected by filtration for intracellular metabolite extraction and quantification. The data used for the heat map were the mean values of three independent experiments. Abbreviations: H6P mean hexose-6-Phosphate which is the sum of Glc6P and Fru6P; FBP means Fructose 1, 6-bisphosphate.

**Figure 6:**
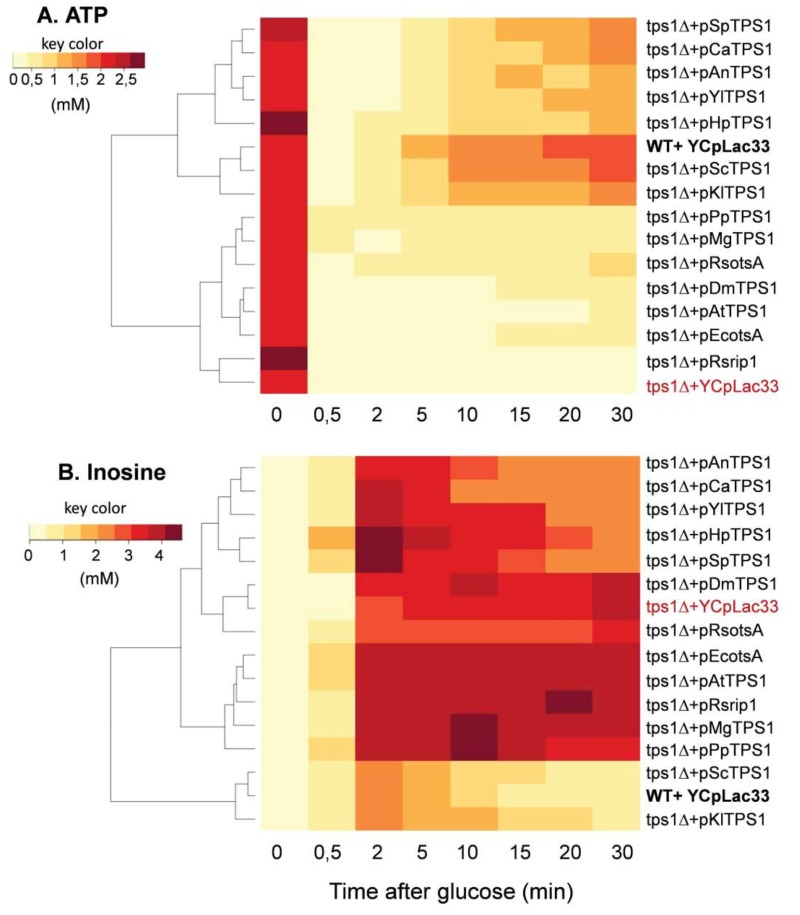
FIGURE 6: Heat map representation of the change of ATP (A) and inosine (B) concentration in *S. cerevisiae tps1 *mutant expressing* TPS1 *from different organisms in response to a glucose pulse. Same procedure as in Fig. 5.

A well-established metabolic defect harbored by a *Sctps1*Δ mutant is the huge accumulation of sugar phosphates, the rapid loss of ATP and the absence of T6P production in response to high glucose 11,15,25,37,38. We therefore investigated the dynamic response of these metabolites to glucose in *Sctps1*Δ transformed with the different *TPS1 *homologues. We chose to present the metabolic data as heat map (Fig.5 and 6) since it provides a better visualization of the dynamic changes that occurred in the different strains and shall facilitate grouping of metabolic patterns according to the *TPS1* homologue which complements the Sctps1Δ mutant. Note that a classical time course representation of the data is reported in Supplementary Figures S6 to S10. We found, as expected, that the metabolic pattern of *Sctps1*Δ complemented with its own gene was identical to that of the wild type strain and this pattern was also found with the *Sctps1*Δ mutant transformed with the*K. lactis TPS1*, which is in line with the close phylogenetic proximity of these two proteins. At the opposite of this pattern, metabolic profiles of the *Sctps1*Δ mutant expressing *TPS1* from *E. coli*, *R. solanacearum *(*otsA *and *rip1*), *A. thaliana* and *D. melanogaster* grouped together and were remarkably similar to that of the *Sctps1*Δ mutant. This second group was characterized by a failure to produce T6P (see also supplementary Figure S6) and by an accumulation of H6P (hexose-6-phosphate) and FBP (fructose-1,6-bisphosphate), which were three and then times higher, respectively, than in wild type in response to glucose. In addition, the recovery of ATP, which has been lost after glucose addition, was dramatically impaired in these transformants (Fig. 6 and Supplementary data in Fig. S8). This deficiency in ATP recovery was concomitant with the accumulation of high levels of inosine, which serves as a sink to avoid the cell to lose nucleotide pools 39.

At variance to the phylogenetic analysis reported in Fig. 1, the metabolic patterns exhibited by *TPS1* homologues from other yeast species and filamentous fungi were rather dispersed into small subgroups, which can be related to either the wild type (group 1) or to the *tps1*Δ profile (group 2). More specifically, the *Sctps1*Δ mutant complemented with *TPS1* homologues from *C. albicans*, *S. pombe*, and *H. polymorpha* and to a lesser extent with *A. nidulans* *TPS1* presented a metabolic pattern in response to glucose which was similar to that of group 1. The most notable differences were, nevertheless, an accumulation of T6P, which was four to ten times lower than in wild type, and a slower recovery of ATP following glucose addition. Probably as a consequence of this slower recovery of ATP, these strains were also characterized by a significant accumulation of inosine, which was slowly remobilized afterwards, leading to their clustering with the *tps1* strain (group 2) for this specific metabolite (Fig. 5 and 6). On the other hand, the metabolic profile harbored by *Sctps1*Δ expressing *TPS1* from *Y. lipolytica*, *P. pastoris* and *M. grisea* is closer to group 2 than group 1, notably with respect to the very low levels of T6P, high accumulation H6P and FBP as well as inosine, upon glucose addition, and as expected a very slow and delayed recovery of ATP. To conclude, these metabolic analyses provided a clear indication that the TPS protein from these different origins is not equally functional and even if the homologous gene can rescue growth on glucose, this does not necessarily imply the rebuilding of the wild type metabolite profile.

### T6P promotes rapid metabolic switch to fermentation

**Figure 7:**
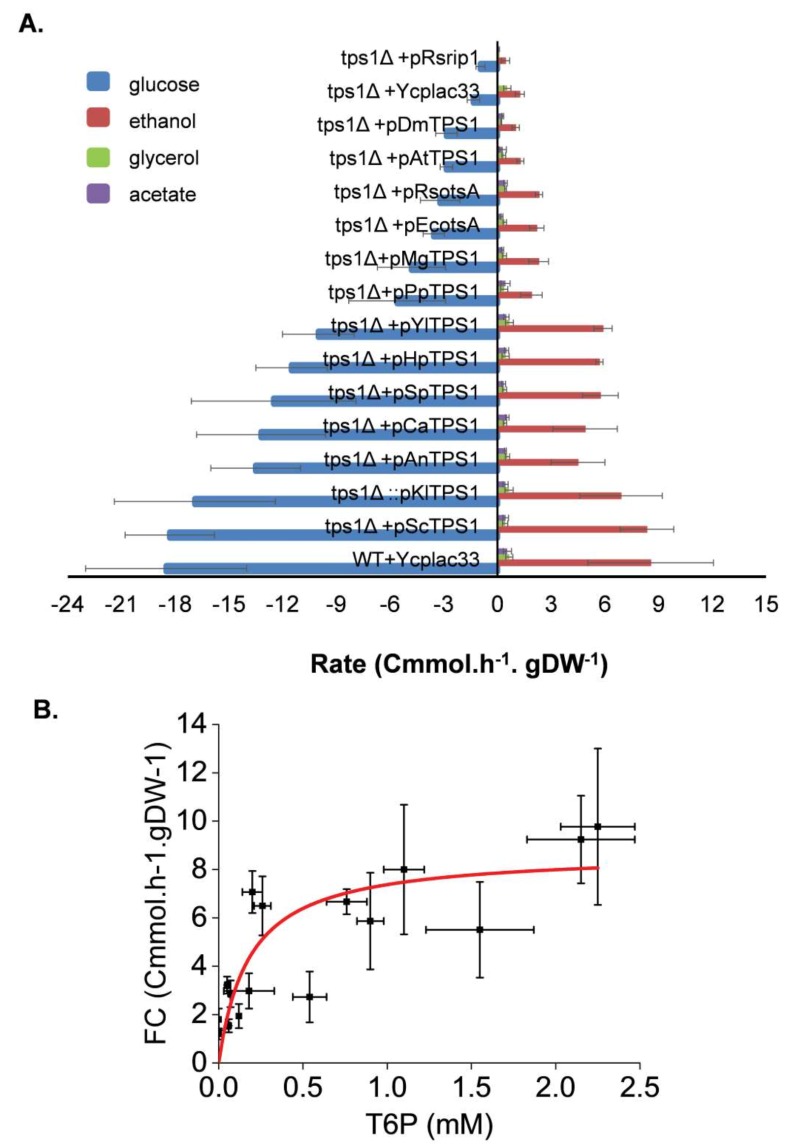
FIGURE 7: Determination of the fermentation capacity of *S. cerevisiae tps1* mutant complemented with *TPS1* from different origins. The experimental design was as described in Fig.3 except that the supernatants of the cultures were taken at 5 min interval after glucose addition for 60 min. Exo-metabolites determined by HPLC were expressed as Cmmol consumed (glucose) or produced (ethanol, acetate and glycerol) per h^-1^ and per g DCW^-1^. Results shown are the mean of three independent experiments with standard deviation bars **(A)**. In **(B)**, Relationship between fermentation capacity (FC) and T6P level. The Origin 2018 software (OriginLab, https://www.originlab.com) was used to fit the data using the non-iterative fitting algorithm (Levenberg-Marquardt) selecting for the Michaelis-Menten or Hill models defined in the software.

When glucose is added to yeast cultivated on a respiratory carbon source, a rapid metabolic shift to fermentation is taking place. This shift is characterized by the production of ethanol and other fermentable byproducts such as glycerol and acetate. The rate at which these products are formed can be related to the fermentation capacity (see 40, for a definition). In agreement with its glucose growth defect phenotype, the loss of *TPS1* function in the yeast *S. cerevisiae* almost eliminated the fermentation of glucose into ethanol (Fig.7A). The reintroduction of the wild type *TPS1* in the *tps1*Δ mutant as well as the *K. lactis* homologous gene fully recovered a wild type fermentation capacity. Also, expression of *TPS1* from other yeast species *S. pombe, C. albicans,* *H. polymorpha* and *Y. lipolytica* as well as that of *A. nidulans* restored the glucose fermentation activity of a Sc*tps1*Δ mutant, but with a 15 to 30% reduced efficiency. By contrast, glucose consumption of the Sc*tps1*Δ mutant expressing the *E. coli*, *R. solanacearum*, *A. thaliana* and *D. melanogaster* *TPS1 *homologue was more than five times lower than in a wild type, and lower sugar consumption was accompanied by weaker production of ethanol. This data indicates that these yeast transformants hardly achieved their metabolic shift towards the fermentation. Likewise, but to a lesser extent, the fermentation activity was reduced by 3 to 4-fold in Sc*tps1*Δ cells transformed with *TPS1* homologues from* P. pastoris *and *M. grisea*. When plotting the fermentation capacity determined in all these yeast strains as a function of T6P measured five min after glucose addition, we obtained a fairly acceptable Michaelis-Menten relationship, allowing to estimate that 50% of the fermentation capacity under these experimental conditions was obtained with about 0.2 mM T6P (Fig. 7B). Likewise, a similar value was obtained by plotting the growth rate on glucose measured in these transformants as a function of T6P (supplementary data, Figure S5B).

### Effective repression of gluconeogenic genes requires T6P 

**Figure 8:**
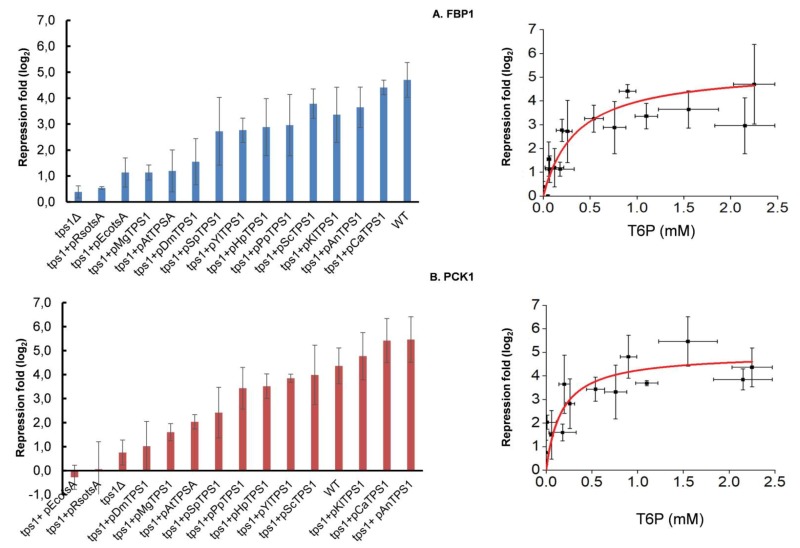
FIGURE 8: Expression level of FBP1 (A) and PCK1 (B) in *S. cerevisiae tps1* mutant complemented with *TPS1* from different organisms. The *Sctps1* strains transformed with YCpL33 plasmid and with *TPS1* homologues were cultivated on YN containing ethanol 2% until 5 units OD_600_. At this time, glucose (10 g/L final) was added and samples (15 OD units) were taken before and 1 h after glucose addition. The transcript levels of *FBP1* and *PCK1* was quantified by RT-qPCR using *TAF10* and *KRE11* as reference genes for normalization. Results shown are the mean of two.

It is well known that glucose triggers the transcriptional repression of the gluconeogenic genes *FBP1 *and *PCK1 *encoding fructose-1,6-biphosphate and phosphoenolpyruvate carboxykinase, respectively. While this repression requires phosphorylation of the sugar 41, the intracellular signal is still unknown 42. Besides the inability of a *S. cerevisiae tps1*Δ mutant to grow on fermentable sugars, it was recently reported that *FBP1 *and *PCK1 *as well as genes of the glyoxylate pathway were not repressed upon glucose addition to this mutant cultivated on a respiratory carbon source 20. Figure 8 shows a hyperbolic relationship that can be drawn by plotting the repression value for *FBP1 *and *PCK1 *genes determined 1 h after glucose addition and the concentration of T6P measured 5 min after the glucose pulse. Therefore, in addition to confirm the finding of these authors, we furthermore showed that the magnitude of the repression of these gluconeogenic genes is very likely dependent on the production of T6P. Interestingly, the T6P concentration that corresponded to half of the calculated maximal fold repression of *FBP1 *and *PCK1 *was in the range of 0.2 - 0.35 mM, which is in the same range of the value found for 50% of the maximal fermentation capacity (Fig. 7).

## DISCUSSION

In this work, we revisited the capability of the *S. cerevisiae* *TPS1* homologous genes from different origins to complement the metabolic defects of a *Sctps1*Δ mutant strain. To realize this comparison in a quantitative manner, we selected *TPS1* homologues harboring sufficient phylogenetic distances, and we cloned them into the centromeric plasmid YCpLac33 under the yeast *PGK1* promoter. A first interesting result from this physiological analysis was to find that even in the absence of detectable T6P, the growth rescue of *tps1*Δ on fermentable sugars was observed. This was demonstrated with *Sctps1*Δ transformed with *TPS1* homologues from bacteria, plant or insect that complemented the glucose-negative defect, albeit levels of T6P in these strains after glucose pulse was below our limit of detection. Should this observation mean that the TPS protein alone is sufficient to restore growth of *Sctps1*Δ on these sugars? We actually propose that a TPS protein that bears intact binding sites for G6P and UDPGlc is absolutely required for yeast to grow on a fermentable sugar, whereas T6P, the product of the reaction catalyzed by this protein, critically promotes sugar fermentation. This proposition is supported by the following data.

Firstly, Delorge *et al*. 43 reported that *TPS* genes from class II from the plant *A. thaliana* were unable to complement the glucose-negative phenotype of *Sctps1*Δ. They ascribed this failure to the lack of these enzymes to have catalytic activity due to the absence of the conserved binding sites for G6P and UDP-Glc in their amino acids sequence 44. Also, Poueymiro *et al*. 34 showed that the catalytic inactive variant of *R. solanacearum* ripTPS^Y154V^ (i.e the replacement of Y at position 154 by V abolishes binding of G6P) has lost its ability to complement a *S. cerevisiae* *tps1*Δ*tps2*Δ mutant strain on glucose and fructose. Consistent with this result, we found that the growth defect of *Sctps1*Δ on glucose was not alleviated by the expression of a catalytically-dead Tps1 from the yeast *S. cerevisiae* (*tps1-156* variant that expresses a variant Tps1 in which the G6P binding is abolished by the replacement of Y at position 156 by V; 35).

Secondly, it was reported that the growth recovery on glucose of the double *tps1*Δ*tps2*Δ mutant strain was more effective than the single *tps1*Δ mutant upon the expression of the *A. thaliana* *TPS2* or *TPS4* gene, which belong to the class I TPS-like proteins 43,45. A similar finding was shown for the *R. solanacerum rip1* gene, which better complemented the glucose negative phenotype of a double *tps1*Δ*tps2*Δ mutant than the single *tps1*Δ mutant 34. It was therefore suggested that the better growth recovery of a *tps1*Δ*tps2*Δ mutant strain expressing these TPS homologs was due to the fact that they could accumulate even very low T6P levels in response to glucose, which was not the case for the single *Sctps1*Δ strain 34,43. From these reports, and with assumption that TPS proteins from these various species are expressed at the same level as that of the wild type yeast, an intriguing question is why they display so significant differences in their catalytic activity. In the case of Tps1 from *A. thaliana* encoded by *AtTPS1*, it was shown that the truncation of its extra N-terminal extension, which is not present in the yeast protein, results in a ten to 40-fold increase of its *in vitro* activity 30. The TPS protein of *R. solanacearum* encoded by *rip1* also harbours an N-terminal extension, whose deletion might result in a similar effect. On the other hand, Tps1 from *P. pastoris*, *A. thaliana* and *D. melanogaster* show a long C-terminal extension which does not exist in any other TPS proteins. The impact of this C-extension on Tps1 activity has not been examined so far. Besides, taking into account that *in vivo S. cerevisiae* Tps1 is present in a complex with three others proteins, namely Tps2, Tps3 and Tsl1 43,46,47, and that the optimal activity of Tps1 likely depends on the interaction with these partners 48, the very low activity of Tps1 from bacteria, plant and insects expressed in yeast might be explained by a failure to properly form this TPS complex. As a conclusion, a more precise structure-function analysis of the yeast Tps1 is awaiting to strengthen our hypothesis of a possible separated function of this protein in growth on fermentable sugar and in purely synthesis of T6P.

The finding that T6P promotes growth resumption on fermentable sugars coincided with the prompt recovery of ATP after its rapid fall following a glucose pulse. The reported inhibition of the major hexose phosphorylating enzyme encoded by *HXK2* 13 could be invoked to account for this effect, because this inhibition would restrict the rate of the upper part of glycolysis which consumes ATP, allowing the lower part of the pathway to regenerate it. However, taken into consideration the inhibition constant of hexokinase II for T6P (i.e. 0.05 to 0.1 mM 13,38), and the assumption that the intracellular glucose concentration is within 0.5 to 2 mM in yeast cells challenged with glucose 49, the hexokinase activity would be maintained inhibited by more than 50% over more than 30 min after the glucose pulse, which would likely penalize the fast resumption of growth and the attendant fermentation activity. On the other hand, the probable absence of this inhibition in *Sctps1*Δ cells expressing *S. pombe*, *Y. lipolityca* or *A. nidulans TPS1* homologues due to their low level of T6P is inconsistent with the fact that the fermentation activity of these yeast strains was only 20 to 30% lower than that of a wild type strain.

As a consequence, and in the light of previous work showing that manipulating the intracellular T6P content up or down has only little impact on the glucose influx into glycolysis 15,25,38,48, one must invoke a complementary mechanism by which T6P promotes rapid growth on fermentable sugars. Interestingly, we found a hyperbolic correlation between fermentation capacity and levels of T6P, which is an indication that this metabolite is indeed a positive effector of fermentation. In addition, this data is consistent with the work of Wu *et al*. 50 which reported a parallel increase in the rate of glucose consumption, ethanol production and the rise of T6P following the short-term (50 to 300 sec) response of carbon-limited chemostat culture to a glucose pulse. Taken these results together, we postulate that T6P is implicated in the short-term Crabtree effect, which can be defined either by the immediate occurrence of aerobic fermentation in response to an excess of sugar to a sugar-limited yeast culture 51,52, or more broadly as the capacity of yeast cells to readily switch from respiration to fermentation in response to glucose 53. Although the molecular mechanism of this metabolic switch is still a matter of debate, it is clear that this switch is intimately dependent on the rate at which glucose enters the cell 40,54,55. Heineman and colleagues 56 have introduced the notion of ‘flux-sensing mechanism’ to account for the fact that these metabolic phenotypes are triggered by a rapid change in sugar influx. Furthermore, they argued that these metabolic shifts require the cell to be able to measure “metabolic flux” through the level of some “flux-sensing” metabolites. FBP has been proposed as a possible flux-sensing molecule based on a good, yet strict, linear correlation between its intracellular levels and the sugar uptake rate 56. In addition, this metabolite has been directly implicated in the Crabtree effect because of its ability to inhibit at physiological concentration (i.e. 2 to 10 mM) the oxidative phosphorylation of isolated mitochondria or permeabilized spheroplasts of yeast 57. However, this latter data is not consistent with the fact that strains harbouring high levels of FBP such as *Sctps1*Δ expressing bacteria, plant or insect TPS protein hardly undergo the fermentation shift upon glucose addition. We therefore propose that T6P is a better flux-sensing molecule than FBP because it is metabolically located closer to the glucose uptake than FBP, it interacts at the gate of the glucose entrance through its action on hexokinase and it exhibits a Michaelis-Menten relationship with fermentation activity. The same flux-sensing function of T6P in glucose repression could be also invoked based on the fact that repression of the gluconeogenic genes *FBP1* and *PCK1* is strictly dependent on T6P. Interestingly, the concentration of T6P that corresponded to 50% of the maximal glucose repression effect is in the same range (i.e. 0.2 - 0.3 mM) as the one estimated to reach 50% of the optimal fermentation activity, which suggests that the molecular targets of this metabolite are the same for these two processes.

The proposed role of T6P as a sugar signaling molecule in yeast is convergent with its assigned function in plants as a sensor of sucrose to integrate carbon assimilation with plant growth 58,59. This integration is obtained by the inhibition of the SNF1-related protein kinase by T6P. Whether this inhibition is direct or indirect is still unclear but, whatsoever the exact mechanism, this action of T6P overcomes the antagonistic effects of this kinase on the anabolic processes and growth in response to high level of sugars 60. In yeast, the Snf1 kinase is essential in the derepression of gluconeogenic genes and in respiration 61. However, contrary to expectation, this kinase is insensitive to T6P 20. A common trait of yeast mutants harboring negligible levels of T6P upon glucose addition is their very lengthy recuperation of ATP, which could be explained not solely by a lack of hexokinase inhibition but also by the absence of a stimulatory action on downstream metabolic targets. In a previous work, we showed that the purine salvage pathway (PSP) was important in the control of ATP homeostasis under transition from respiration to fermentation and that the rebuilding of ATP shortly after a glucose pulse was largely attributed to the reassimilation of inosine 39. Therefore, enzymes of the PSP can be potential targets of T6P. It is also conceivable that the failure of glucose repression of *FBP1* and *PCK1* is a consequence of the very low levels ATP in these strains which harbor low to negligible production of T6P.

## MATERIALS AND METHODS

### Yeast strains, plasmids and transformation 

The *Saccharomyces cerevisiae tps1*Δ and *tps2*Δ mutants were derived from the CEN.PK133-5D *ura3-52 *strain 28 and have been described elsewhere 15,62. The *tps1*Δ strain was used as the host for transformation with the YCplac33 plasmid 63 bearing *TPS1 *from various origin (*Saccharomyces cerevisiae*, *Drosophila melanogaster*, *Arabidopsis thaliana*, *Escherichia coli*, *Aspergillus nidulans*, *Hansenula polymorpha*, *Pichia pastoris*, *Magnaporthe grisea*, *Yarrowia lipolytica*, *Kluyveromyces lactis*, *Candida albicans*, *Schizosaccharomyces pombe *and *Ralstonia solanacearum*) under the control of the *S. cerevisiae PGK1 *promoter. The *TPS1 *coding sequence from these organisms was amplified by PCR using 5’ and 3’ primers listed in Table S1. The PCR fragments were cloned into the YCplac33 plasmid using the In-Fusion system (Clontech), except the ones from *M. grisea *and *P. pastoris, *for which exons were amplified and assembled by using the NEBuilder® HiFi DNA Assembly system (New England Biolabs). All plasmid constructs bearing the *TPS1 *homologues are listed in Table 3 and were verified by DNA sequencing. The Lithium acetate procedure described in 64 was used to transform *S. cerevisiae tps1*Δ mutant with the different plasmids.

**Table 3:** List of plasmids used in this study.

	**Name**	**Description**	**Reference**
	**YCplac33**	CEN origin, URA3 marker	61
	**pScTPS1**	YCplac33 backbone with *S. cerevisiae TPS1* gene	This study
	**pDmTPS1**	YCplac33 backbone with *D. melanogaster Tps1* gene	This study
	**pAthTPS1**	YCplac33 backbone with *A. thaliana TPS1* gene	This study
	**pAnTPS1**	YCplac33 backbone with *A. nidulans tpsA* gene	This study
	**pHpTPS1**	YCplac33 backbone with *H. polymorpha TPS1* gene	This study
	**pPpTPS1**	YCplac33 backbone with *P. pastoris TPS1* gene	This study
	**pMgTPS1**	YCplac33 backbone with *M. grisea TPS1* gene	This study
	**pYlTPS1**	YCplac33 backbone with *Y. lipolytica TPS1* gene	This study
	**pKlTPS1**	YCplac33 backbone with *K. lactis TPS1* gene	This study
	**pCaTPS1**	YCplac33 backbone with *C. albicans TPS1* gene	This study
	**pSpTPS1**	YCplac33 backbone with S*. pombe TPS1* gene	This study
	**pEcotsA**	YCplac33 backbone with E*. coli otsA* gene	This study
	**pRsotsA**	YCplac33 backbone with R*.solanacearum otsA* gene	This study
	**pRsrip1**	YCplac33 backbone with R*.solanacearum rip1* gene	This study

### Culture conditions

Unless otherwise stated, yeast cells were cultivated on a YN synthetic medium (17 g of yeast nitrogen base without amino acids and 5 g of ammonium sulfate per liter) buffered at pH 5.5 with 13.5 g/l succinic acid and 6.5 g/l NaOH**, **with 2% galactose as carbon source and complemented with 100 mg/l uracil. To verify that the *tps1*Δ mutant has not undergone phenotypic or genetic ‘reversion’ that allows growth on glucose 14,65, we streaked this strain on a YN fructose 2% complemented with uracil. Under this condition, no colonies were seen after at least 5 days of incubation at 30°C, whereas several ‘colonies’ arose after 2 days on YN glucose + uracile agar plates. Unless otherwise stated, growth of the mutant and transformants was carried out in baffled 1 L Erlenmeyer flasks containing 200 ml of culture medium at 30°C on a rotary shaker at 200 rpm. The carbon source (glucose, galactose, raffinose) was added at 2% (w/v) except trehalose which was added at 1% (w/v). Growth on Petri dish plates was carried out in YN synthetic medium containing glucose or galactose at 2% (w/v) and supplemented with agar 2% (w/v).

### Glucose pulse and intracellular metabolites determination 

The glucose pulse experiment was performed as described in a previous report 39. Briefly, the yeast cells were cultivated in YN containing 1% trehalose and buffered to pH 5.5 using 50 mM potassium phthalate instead of Na-Succinate until they reached OD_600_ 5 units (i.e. around 1 g dry mass per liter). Then, they were harvested, washed once with water at room temperature and resuspended in YN medium for 30 min at 30°C. Then, glucose was added to a final concentration of 10 g/l using a concentrated stock solution of 50% (w/v). Samples (5 ml) were harvested at the indicated timepoints in the corresponding figures (Fig 4 to 6) by rapid filtration on 0.45 μm porosity nitrocellulose filter and immediately quenched in 3 - 5 ml of 75% hot ethanol, followed by 4 min of incubation in a water bath set at 80°C according to 66. After extraction, removal of ethanol by evaporation and resuspension of the dry pellet in 0.5 ml of sterile water, the metabolites were quantified in an ICS-3000 system equipped with an automatic eluent (KOH) generator system (RFIC, Dionex), an autosampler (AS50, Dionex), a photo diode array detector (Ultimate 3000, Dionex), a conductivity detector (part of ICS-3000, Dionex) and a mass-sensitive detector (MSQ Plus, Thermo Scientific) running in ESI mode (nitrogen pressure was 90 psi (1 psi=6.9 kPa), capillary voltage was 3.5 kV and probe temperature was 450°C), according to 39. The concentration of intracellular metabolites is expressed in mM taking into account the assumption that 1 g of dry cell wall contains 2 ml of cell sap 49.

### Determination of gene expression levels by RT-qPCR 

Quantification of *TPS1*, *URA3 FBP1 *and *PCK1 *transcripts was performed from two independent biological cultures. Yeast cells (about 15 OD_600_ units) were collected by centrifugation (3000 rpm, 4°C, 2 min), followed by a washing step with 1 ml of sterilized water. The cell pellets were immediately frozen in liquid nitrogen and stored at - 80°C. Cell disruption was performed with a Tissue Lyser system (Qiagen) at 30 Hz during 3 minutes. Total RNA was isolated according to RNeasy Total RNA Isolation Kit Manual (Qiagen) and concentrations of RNA samples were calculated by using NanoDrop 2000 UV-Vis spectrophotometer (Thermo Scientific, USA). Quality assessment of RNA samples was performed by Agilent RNA 6000 Nano kit and 2100 Bioanalyzer (Agilent Technologies, USA). Complementary DNA was synthetized with 1 μg of total RNA using the iScript cDNA Synthesis Kit (Bio-Rad). The absence of genomic DNA in RNA samples was checked by qPCR before cDNA synthesis (minus RT control). A blank (No Template Control) was also incorporated in each assay. The Quantitative PCR (qPCR) was performed using the Sso Advanced Universal SYBR Green system (Bio-Rad), 4 μl of cDNA 10 times diluted and 2 μl of primers (2 mM). The PCR program comprised an initial denaturation step of 1 minute at 95°C and amplification by 40 cycles of 10 sec at 95°C, 45 sec at 54°C in a Bio-Rad MyIQ cycler. The *TAF10 *and *KRE11 *genes were used as reference genes for normalization, following the recommendation of Teste *et al*. 67 for accurate transcripts measurement. Primer sequences used for RT- qPCR are provided in Table S2.

### Assay of Trehalose-6P synthase (Tps1) activity 

The activity of TPS was determined in cell crude extracts according to the procedure described in 68. Briefly, yeast cells (about 10 mg dry mass or 50 OD_600_ units) were collected by centrifugation (3 min at 2000*g*, 4°C) and washed with cold sterile water. Crude extract was performed by breaking yeast cells in 0.5 ml extraction buffer (20 mM Hepes, pH 7.1 containing 1 mM EDTA, 100 mM KCl, 1mM DTT, and 1mM PMSF) with 0.5 g glass beads (0.5 mm diameter; BioSpec 11079105) on a vortex set at full speed. The vortexing was done 6 times for 30 sec with 30 sec intervals where tubes were kept on ice. The assay was realized at 42°C in the presence of 10 mM Glucose-6-P/3 mM Fructose-6-P and 5 mM UDP-glucose in 20 mM Hepes buffer, pH 7.1, 5 mM MgCl2, 1 mM EDTA, 100 mM KCl. The reaction was stopped by heat (5 min at 80°C). The Tps1 activity was determined either by the amount of UDP released or by the amount of trehalose-6-phosphate (T6P) produced and determined as intracellular metabolites by LC-MS as described above. Proteins were quantified with the Bradford reagent (Bio-Rad protein assay dye reagent concentrate, 500-0006).

### Analytical methods 

Glucose, ethanol, acetate and glycerol were determined in the medium supernatant by withdrawing 1 ml from the YN glucose cultures at the desired times. Samples were centrifuged at 13,000 rpm for 5 min in a bench-top centrifuge (Eppendorf 5415D), supernatants were filtered through a 0.45 μm syringe filter, and stored at -20 °C until further analysis. Quantification was carried out using High performance liquid chromatography (HPLC) on an Ultimate 3000 system (Dionex, Sunnyvale, USA). The HPLC system was equipped with a cation-exchange column (Aminex HPX-87H - 300 x 7.8 mm, 9 μm, Biorad), an autosampler (WPS-3000RS, Dionex), a RI detector (RID 10A, Shimadzu), and an UV/VIS detector (SPD-20A, Shimadzu). The mobile phase was 1.25 mM H2SO4 at a flow rate of 0.5 ml/min. Column temperature was held at 35°C. Glycogen and trehalose content were measured by glucose colorimetric assay after Na2C03 treatment of the whole cell as described by 69.

## SUPPLEMENTAL MATERIAL

Click here for supplemental data file.

All supplemental data for this article are also available online at http://microbialcell.com/researcharticles/trehalose-6-phosphate-promotes-fermentation-and-glucose-repression-in-saccharomyces-cerevisiae/.
