# Supplementary material for: Trehalose-6-phosphate promotes fermentation and glucose repression in Saccharomyces cerevisiae
Source: Microb Cell. 2018 Oct 1;5(10):444–59. doi: 10.15698/mic2018.10.651 (PMC6206404; doi:10.15698/mic2018.10.651)
Supplement: Supplementary file 1 [file mic-05-444-s01.pdf]

Table S1: Oligonucleotides used for PCR amplification of the different *TPS1* genes.

Name	Sequence
**ScTPS1Fw**	CGACTCTAGAGGATCCATGACTACGGATAACGCTA
**ScTPS1Rv**	CGGGCCCCCCCTCGAGTCAGTTTTTGGTGGCAGA
**DmTPS1Fw**	TAGAGGATCCCCCGGGATGCCCGACACGGAAAT
**DmTPS1Rv**	CGGGCCCCCCCTCGAGTCACACCTCCAGATTGTTCG
**AtTPS1Fw**	AGATCTCGACTCTAGAATGCCTGGAAATAAGTACAACT
**AtTPS1Rv**	ATTCCTGCAGCCCGGGTTAAGGTGAGGAAGTGGTGT
**EcOTSAFw**	TAGAGGATCCCCCGGGATGAGTCGTTTAGTCGTAG
**EcOTSARv**	CGGGCCCCCCCTCGAGCTACGCAAGCTTTGGAAAG
**AnTPSAFw**	CGACTCTAGAGGATCCATGCCCGGCGTTGAGAA
**AnTPSARv**	CGGGCCCCCCCTCGAGCTACTGTGACGAAGTCTCTAGCTTTGA
**HpTPS1Fw**	CGACTCTAGAGGATCCATGGTCAAAGGTAATGTTATAG
**HpTPS1Rv**	CGGGCCCCCCCTCGAGTCACACTCTCGTCAATTCTTTC
**YlTPS1Fw**	CGACTCTAGAGGATCCATGCCCAACGTGCTGGT
**YlTPS1Rv**	CGGGCCCCCCCTCGAGTCAACACCGCTTCAACTCG
**KlTPS1Fw**	CGACTCTAGAGGATCCATGGTTAATCAAGACATTAGTAAGCT
**KlTPS1Rv**	CGGGCCCCCCCTCGAGTCAGTTGTTTGACGATCCTAAT
**CaTPS1Fw**	CGACTCTAGAGGATCCATGGTTCAAGGAAAAGTCTTG
**CaTPS1Rv**	TTGATATCGAATTCCTGCAGCTAGTCCCTCAAACTCTTTTG
**SpTPS1Fw**	CGACTCTAGAGGATCCATGTCGGATGCTCATGATA
**SpTPS1Rv**	CGGGCCCCCCCTCGAGTTACGAGCTAGAATTCATCGA
**RsOTSAFw**	CGACTCTAGAGGATCCATGAGCCGGCTGATCGTG
**RsOTSARv**	CGGGCCCCCCCTCGAGCTAGCGGGCCGATGCG
**RsRIPTPSFw**	CGACTCTAGAGGATCCATGCGCCCGATTATTTCCA
**RsRIPTPSRv**	CGGGCCCCCCCTCGAGCTACGCCTGCGTGAGAC
**MgE1Fw**	ACAAGATCTCGACTCTAGAGATGGGCAGCGTTGAAGAC
**MgE1Rv**	AGGATCGAGTTGGCAAACCCGTTGTAGTG
**MgE2Fw**	GGGTTTGCCAACTCGATCCTCTGGCCAC
**MgE2Rv**	TGGCGCAGACAAGATACGTGAGCATGAGCTC
**MgE3Fw**	CACGTATCTTGTCTGCGCCAACAACCCC
**MgE3Rv**	CTTGATATCGAATTCCTGCATCAGTTTCCCTCCGTCTTGTTG
**PichiaE1Fw**	GATCTCGACTCTAGAGGATCCATGGTGGAAGGAAATGTTC
**PichiaE1Rv**	TTGCTAGGCCTTGTGAGTTCCTTGAC
**PichiaE2Fw**	TCACAAGGCCTAGCAACATCAGCAGTTC
**PichiaE2Rv**	GTACCGGGCCCCCCCTCGAGTTATGTGTTCTCTGCTAGTATTTC

Table S2: qRT-PCR primers used in this study.

Gene	Forward	Reverse	Reference
**TAF10**	ATATTCCAGGATCAGGTCTTCCGTAGC	GTAGTCTTCTCATTCTGTTGATGTTGTTGTTG	[57]
**KRE11**	AACTGGTTCTGTTACCCAAATCAACTCAAC	AACGCTTCAATGTGACTTCTGTTTCCC	[57]
**PCK1**	CTTCCTAGCCTTGCACCCTA	CATTGGCTAACGAACCATCA	[19]
**FBP1**	AATGGTAGCCGCTTGCTATG	AATTCGCCCAAGTTTGTGTC	[19][
**URA3**	TGACATTGCGAAGAGCGACAAAG	CCAATGCGTCTCCCTTGTCATCTA	This study
**ScTPS1**	AATCAACGGTCAGTTCGGTA	CAAACAAACATCGCTCACAG	This study
**DmTPS1**	CGCCCGAAATTAAGAATGTA	ATGCCAACCATCTTCTTGAC	This study
**AtTPS1**	TGCATCAGTTGATGTTGTCC	CAATGCACAAGACGTAATCG	This study
**EcotsA**	CGTTCTTGTTCTTTCGCAAT	CATAGTCAATGCACGATCCA	This study
**AnTPS1**	AATTTGAGGGCTGTTGTGAA	AATTCACCGATTTGTGGAGA	This study
**HpTPS1**	GACGGAATGAATTTGGTCAG	AGCGCCATTTAATGACTGAG	This study
**YlTPS1**	ACTTCAACGAGCTGATTGCT	ATCCGTGCTTCTCAGTTTGA	This study
**KlTPS1**	TTACCCAATGGTGTCGAGTT	ACAGCATCTTGCTTCAAACC	This study
**CaTPS1**	ACCATTCCCATCATCAGAAA	AAATGACGGGCGTAATCATA	This study
**SpTPS1**	ACCCGTTAGAAACGAAATCC	CATGCAGACAAAAAGTGACG	This study
**RsotsA**	ATGTGCAGAGCTACCAGCAG	CTTGTTGATGTAGCGGATCG	This study
**Rsrip1**	GGGTCCACGACTACCATCTC	CAGCGGAATATGGTTGAAGA	This study
**MgTPS1**	TGAGAGGTACGTCTTCAAGTACA	ATCTTGGTCTTTGAGCCTTC	This study
**PpTPS1**	CCAATTATGCCCGACTATTG	CCTCCAAAGAAACGCTCATA	This study

Conserved binding sites for Glucose 6-phosphate and UDPglucose among the Tps1 proteins from different organisms

**Origin**	**Glc-6-phosphate binding residues***				
	**R9**	**W40**	**Y76**	**W85**	**R300**
***S. cerevisiae* Tps1**	VVSNRLPVT	KWFGWPGLE	ADLHYNGFS	NSILWPLFH	AVPSRGDVE
***D. melanogaster* Tps1**	VVSNRLPFV	LWVGWSGIH	FDSYYNGCC	NKIFWPLFH	SVPSRTDVK
***A. thaliana* TpsA**	LISNRLPIT	RWIGWAGVN	VHQYYNGYC	NNILWPLFH	AVPTRTDVP
***A. nidulans* TpsA**	LVSNRLPIT	QWYGWPGLE	ADRHYNGFS	NSILWPLFH	AVPSRQDVE
***H. polymorpha* Tps1**	VVSNRIPVT	RWFGWPGMS	ADLHYNGFS	NSILWPLFH	AVPSRGDVE
***P. pastoris* Tps1**	VISNRVPVT	QWFGWPGLE	AELHYNGFS	NSILWPLFH	AVPSRGDVE
***M. grisea* Tps1**	LISNRLPIT	QWYGWPGLE	ADRHYNGFA	NSILWPLFH	AVPSRQDVE
***Y. lipolytica* Tps1**	VISNRLPVT	QWFGWPGLE	ADLHYNGFS	NSILWPLFH	AVPSRGLVE
***K. lactis* Tps1**	VISNRLPVT	QWYGWPGLE	ADLHYNGFS	NSILWPLFH	AVPSRGDVE
***C. albicans* Tps1**	VVSNRIPVT	QWYGWPGLE	ADLHYNGFS	NSILWPLFH	AVPSRGDVE
***S. pompbe* Tps1**	LVSNRLPIT	QWLGWCGQE	ADRHYNGFS	NSILWPLFH	AVPSRQDVE
***E. coli* OtsA**	VVSNRIAPP	LWFGWSGET	LDEYYNQFS	NAVLWPAFH	APTSRGDVQ
***R. solanacearum* OtsA**	VVSNRVAPI	VWFGWSGEI	YDQYYRGFA	NATLWPVFH	APTSRQDVQ
***R. solanacearum* Rip**	VVSNRLIDP	LWFGWSGKT	YEGYYAGFS	NSVLWPIFH	AAPTRQSIP

**Origin**	**UDP binding residues***					
	**G22**	**D130**	**H154**	**R262**	**D361**	**E369**
***S. cerevisiae* Tps1**	AMSSGGLVT	IWVHDYHLM	GWFLHTPFP	VGVDRLDYI	SSTRDGMNL	LVSYEYIAC
***D. melanogaster* Tps1**	RASAGGLVT	VWIHDYHLM	AFFLHIPFP	LGVDRLDYT	TPLRDGMNL	LVAKEFVAC
***A. thaliana* TpsA**	EISAGGLVS	VWCHDYHLM	GWFLHTPFP	LGVDRLDMI	TSLRDGMNL	LVSYEFVAC
***A. nidulans* TpsA**	SMSSGGLVS	IWVHDYHLM	GFFLHTPFP	VGVDRLDYI	SSTRDGMNL	LVSYEYIAT
***H. polymorpha* Tps1**	TMSSGGLVT	IWVHDYHLM	GFFLHTPFP	IGVDRLDYI	SSTRDGMNL	LVSYEYIAC
***P. pastoris* Tps1**	NMSSGGLVT	VWIHDYHLM	GFFLHTPFP	VGVDRLDYI	SSTRDGMNL	LVSYEYIAC
***M. grisea* Tps1**	SMSSGGLVT	IWVHDYHLM	GFFLHTPFP	VGVDRLDYI	SSTRDGMNL	LVSYEYIAT
***Y. lipolytica* Tps1**	TMSSGGLVT	VWVHDYHLM	GFFLHTPFP	VGVDRLDYI	SSTRDGMNL	LVSYEYVAC
***K. lactis* Tps1**	SMSSGGLVT	VWVHDYHLM	GWFLHTPFP	IGVDRLDYI	SSTRDGMNL	LVSYEYISC
***C. albicans* Tps1**	SMSSGGLVT	IWVHDYHLM	GFFLHTPFP	VGVDRLDYI	SSTRDGMNL	LVSYEYIAC
***S. pompbe* Tps1**	SMSSGGLVS	IWVQDYHLM	GFFLHTPFP	VGVDRLDYI	TSTRDGMNL	LVSYEYICT
***E. coli* OtsA**	AASAGGLAV	IWIHDYHLL	GFFLHIPFP	FSVERLDYS	TPLRDGMNL	LVAKEYVAA
***R. solanacearum* OtsA**	QASAGGLAV	LWVHDYHLI	GFFLHIPFP	MSVDRLDYS	TPLRDGMNL	LVAKEYVAA
***R. solanacearum* Rip**	RPAAGGLAV	LWVHDYHLI	GFFNHIPLP	IGVDRLDYS	TPVADGMNL	LVAKEYVAA

Figure S1: Conserved binding sites for Glucose 6-phosphate (G6P) and UDP-glucose (UDP-Glc) among the TPS proteins from different organisms. Alignment of the protein sequences was done using the multiple sequence alignment tool Clustal Omega. Conserves residues were identified using *E. coli otsA* as the reference, previously determined by [1].

Full alignment of Tps1 homologues

```
                            1                                                                                                G 100
  TPS_S cerevisiae    (1) ----------------------------------------------------------------------MTTDNAKAQLTSSSGG------NIIVVSNR
      TPS_K lactis    (1) ---------------------------------------------------------------------MVNQDISKLSLNECPG-------SVIVISNR
    TPS_C albicans    (1) ---------------------------------------------------------------------------------MVQG-------KVLVVSNR
  TPS_H polymorpha    (1) ---------------------------------------------------------------------------------MVKG-------NVIVVSNR
    TPS_P pastoris    (1) ---------------------------------------------------------------------------------MVEG-------NVLVISNR
    TPS_A nidulans    (1) -------------------------------------------------------------------------MPGVEKSSQNES-------RLLLVSNR
      TPS_M grisea    (1) ------------------------------------------------------------------------MGSVEDETALPSGG------RLLLISNR
       TPS_S pombe    (1) ------------------------------------------------------------------------MSDAHDTIKSLTGDASNSR-RLIVVSNR
  TPS_Y lipolytica    (1) -----------------------------------------------------------------------------------MP-------NVLVISNR
    TPS_A thaliana    (1) MPGNKYNCSSSHIPLSRTERLLRDRELREKRKSNRARNPNDVAGSSENSENDLRLEGDSSRQYVEQYLEGAAAAMAHDDACERQEVRPYNRQRLLVVANR
RIP_R solanacearum    (1) --------MRPIISTPAGTSSLAIPDTPGADEAQRADPFQPASRPRSRHPQFERLPSPPVRTRSQELRAARLRRCASDVSATPEAR-----PRLVVVSNR
        TPS_E coli    (1) -----------------------------------------------------------------------------------MS-------RLVVVSNR
TPS_R solanacearum    (1) -----------------------------------------------------------------------------------MS-------RLIVVSNR
TPS_D melanogaster    (1) -------------------------------------------------------------------------MPDTEIIVTNAGE-PSTKASLIVVSNR

                          101                        U                   G                                             G    200
  TPS_S cerevisiae   (25) LPVT-IT--KNSST--GQ--YEYAMSSGGLVTALEGLKKTYTFKWFGWPGLE-------IPDDE--KDQVRKDLLEKFNAVPIFLSDEIADLHYNGFSNS
      TPS_K lactis   (25) LPVT-IK--KDEKT--GE--YEYSMSSGGLVTALQGLKKSTTFQWYGWPGLE-------VPDED--KAKVKRELLEKFNAIPIFLSDEVADLHYNGFSNS
    TPS_C albicans   (13) IPVT-IK--RLDN---GS--YDYSMSSGGLVTALQGLKKTTEFQWYGWPGLE-------IPEDE--QTKVNDELKSKFNCTAIFLSDTIADLHYNGFSNS
  TPS_H polymorpha   (13) IPVT-IK--KTEDDENGKSRYDYTMSSGGLVTALQGLKN--PFRWFGWPGMS-------VDSEQG-RQTVERDLKEKFNCYPIWLSDEIADLHYNGFSNS
    TPS_P pastoris   (13) VPVT-IK--KGSD---GN--YQYNMSSGGLVTALQGLKQ--KFQWFGWPGLE-------VPAID--QEKVNSDLKEKFNCFPIYLSDEIAELHYNGFSNS
    TPS_A nidulans   (21) LPIT-IK--RSED---GK--YDFSMSSGGLVSGLSGLSKTTTFQWYGWPGLE-------VPEEE--IPTLKNRLKEEYNAIPVFIDDELADRHYNGFSNS
      TPS_M grisea   (23) LPIT-IK--RSDD---GQ--YSFSMSSGGLVTGLSGLAKTTSFQWYGWPGLE-------VPDAE--AGPVVQRLKNEYGAHPVFVDDELADRHYNGFANS
       TPS_S pombe   (28) LPIT-IK--RKDN---GT--YDFSMSSGGLVSALSGLKKLMTFQWLGWCGQE-------IPEDE--KPMIIQRLQDECSAIPVFLDDETADRHYNGFSNS
  TPS_Y lipolytica   (11) LPVT-IS--REED---GT--YKYTMSSGGLVTALSGLKQSTTFQWFGWPGLE-------IPEKD--KPRLINDLETMFSCVPVFMDDDLADLHYNGFSNS
    TPS_A thaliana  (101) LPVSAVR--RGED----S--WSLEISAGGLVSALLGVKE-FEARWIGWAGVN-------VPDEVG-QKALSKALAEK-RCIPVFLDEEIVHQYYNGYCNN
RIP_R solanacearum   (88) LIDP------------------QRPAAGGLAVALGDMMRDTDGLWFGWSGKT-------VDEPD--TACVRTEPFGRTTLAQVDLGRTDYEGYYAGFSNS
        TPS_E coli   (11) IAPP--------D--------EHAASAGGLAVGILGALKAAGGLWFGWSGET--------GNED---QPLKKVKKGNITWASFNLSEQDLDEYYNQFSNA
TPS_R solanacearum   (11) VAPI----------------AEGQASAGGLAVGVYDALRDTGGVWFGWSGEI-------APGAS---GEPSIAAKGAITFATVGLSRRDYDQYYRGFANA
TPS_D melanogaster   (27) LPFVLIRDPKTDE-------LERRASAGGLVTAVCPVVIKGSGLWVGWSGIHLKDPNEAIPESNPNDQTPTAGLKSE-QVVSVNIDSKIFDSYYNGCCNK

                      201   G                                                            U                                 300
  TPS_S cerevisiae  (109) ILWPLFHYH--PGEINFD-----ENAWLAYNEANQTFTNEIAKTMN------HND-----LIWVHDYHLMLVPEMLRVKI-HEKQLQNVKVGWFLHTPFP
      TPS_K lactis  (109) ILWPLFHYH--PGEITFD-----DTAWLAYNEANMAFADEIEGNIN------DND-----VVWVHDYHLMLLPEMIRQRV-IAKKLKNIKIGWFLHTPFP
    TPS_C albicans   (96) ILWPLFHYH--PGEMNFD-----ENAWAAYIEANKKFALEIVKQVN------DDD-----MIWVHDYHLMLLPEMLRQEI-GNKK-KNIKIGFFLHTPFP
  TPS_H polymorpha  (100) ILWPLFHYH--PGEMNFD-----EIAWAAYLEANKLFCQTILKEIK------DGD-----VIWVHDYHLMLLPSLLRDQL-NSKGLPNVKIGFFLHTPFP
    TPS_P pastoris   (94) ILWPLFHYH--PGEMNFD-----EAAWAAYIEANRQFCKVIVGKIA------DND-----LVWIHDYHLMLLPQMLRRQI-DEQGLKNVRLGFFLHTPFP
    TPS_A nidulans  (104) ILWPLFHYH--PGEITFD-----ESAWEAYKEANRLFAQAVASQVQ------DGD-----LIWVHDYHLMLLPEMLREEIGNTKK--NIKIGFFLHTPFP
      TPS_M grisea  (106) ILWPLFHYH--PGEITFD-----ESAWSAYKEVNRLFAQTVVKDVQ------DGD-----MIWVHDYHLMLLPEMLREEIGDSKK--NVKIGFFLHTPFP
```

```
      TPS_S pombe  (111) ILWPLFHYH--PGEINFD-----EENWEAYRAANYAFAEAIVKNLQ------DGD-----LIWVQDYHLMVLPQMLRELIGDKFK--DIKIGFFLHTPFP
  TPS_Y lipolytica  (94) ILWPLFHYH--PGEMNFD-----QVAWEAYTQANRLFAKKVASIVK------PGD-----IVWVHDYHLMLLPEMLREECENNSALDGLKIGFFLHTPFP
    TPS_A thaliana (183) ILWPLFHYLGLPQEDRLATTRSFQSQFAAYKKANQMFADVVNEHYE------EGD-----VVWCHDYHLMFLPKCLKEY--NSK----MKVGWFLHTPFP
RIP_R solanacearum (161) VLWPIFHER--VKWADLN-----PAYFKAYEAVNRILASHLRPMLR------DSD-----ILWVHDYHLIPFAQALRAL--GCTQ----RMGFFNHIPLP
        TPS_E coli  (84) VLWPAFHYR--LDLVQFQ-----RPAWDGYLRVNALLADKLLPLLQ------DDD-----IIWIHDYHLLPFAHELRKR--GVNN----RIGFFLHIPFP
TPS_R solanacearum  (85) TLWPVFHYR--VDLARYE-----RQEYHGYRRVNTQFAHQLKALVQ------PDD-----ILWVHDYHLIPFAAECRAL--GLRN----RIGFFLHIPFP
TPS_D melanogaster (119) IFWPLFHSM--PGRANFG-----GEHWHDYVTVNKHFAVRTIEALEKCLAKNQGSEKSPPIVWIHDYHLMLAANWVREHA-EEKN-LPCRLAFFLHIPFP

                         301                                                                                                400
  TPS_S cerevisiae (190) SSEIYRILPVRQEILKGVLSCDLVGFHTYDYARHFLSSVQRVL-N--VNTLPNG--VEYQGRFVNVGAFPIGIDVDKFTDGLKKESVQ--KRIQQLKETF
      TPS_K lactis (190) SSEIYRILPVRQEILKGVLSCDLIGFHTYDYARHFLSAVQRIL-N--VNTLPNG--VEFDGRFVNVGAFPIGIDVETFTEGLKQDAVI--KRIKELKESF
    TPS_C albicans (176) SSEIYRILPVRKEILEGVLSCDLIGFHTYDYARHFISSVSRIVPN--VSTLPNG--IKYQGRSISIGAFPIGIDVDNFIDGLKKDSVV--ERIKQLKSKF
  TPS_H polymorpha (181) SSEIYRILPVRKEILEGVLSCDLIGFHTYDYVRHFLSSVERIL-K--LRTSPQG--VVYNDRQVTVSAYPIGIDVDKFLNGLKTDEVK--SRIKQLETRF
    TPS_P pastoris (175) SSEIYRILPVRKEILEGVLSCDLIGFHTYDYARHFTSSVERIL-G--CRTTPTG--LTYGSHNVSVGAYPIGIDVDKFTTGIKQHLVQ--ERIHSLQKRF
    TPS_A nidulans (184) SSEIYRILPVRNELLLGLLHCDLIGFHTYDYTRHFLSACSRLL-G--LPTTPNG--IEFQGKIIACGAFPIGIDPEKFKEGLKKEKVQ--KRIATLEQKF
     TPS_M grisea  (186) SSEIYRILPVRQALLQGVLHCDLLGFHTYDYARHFLSSCSRIL-S--APTTPNG--VQFAGRFVTVGAFPIGIDPEKFVEGLQKPKVQ--QRIAALTRKF
      TPS_S pombe  (191) SSEIYRVLPVRNEILEGVLNCDLVGFHTYDYARHFLSACSRIL-N--LSTLPNG--VEYNGQMVSVGTFPIGIDPEKFSDALKSDVVK--DRIASIERRL
  TPS_Y lipolytica (176) SSEIYRILPVRKEVLTGVLSCNLIGFHTYDYARHFLSSVSRIL-D--LETMPNG--TYYKGRHVVVGAFPIGIDVNKFLEGCKRPAVQ--ERIAQLQDKF
    TPS_A thaliana (266) SSEIHRTLPSRSELLRSVLAADLVGFHTYDYARHFVSACTRIL-G--LEGTPEG--VEDQGRLTRVAAFPIGIDSDRFIRALEVPEVI--QHMKELKERF
RIP_R solanacearum (237) SPDVIRRIPQHRQLMRALASYDLVGMQSPRDVLN----LQRYW-----DAENIA--AQSERRHDSVHAFPIGIDVESLRALVPSPASQ--AVIDEVRGAA
        TPS_E coli (160) TPEIFNALPTYDTLLEQLCDYDLLGFQTENDRLAFLDCLSNLT----RVTTRSAKSHTAWGKAFRTEVYPIGIEPKEIAKQAAGPLP---PKLAQLKAEL
TPS_R solanacearum (161) SPEILTTIPPHEELMRALCAYDLLGFQTETDRVAFYDYIEREARGYIENKEHNG-PVHAYGNTLRAEVYPIGVHPDEIARQAVSSLARRNPFAREADASG
TPS_D melanogaster (210) PWDIFRLLPWSDEILQGMLGCDLVGFHIQDYCLNFVDCCQRNL-G--CRVDRNNLLVEHGGRTVRVRPLPIGIPYERFVN-LATTAP----KVLKTSK—

                         401                                                    G                                           500
  TPS_S cerevisiae (283) -KGCKIIVGVDRLDYIKGVPQKLHAMEVFLNEHPEWRGKVVLVQVAVPSRGDVEEYQYLRSVVNELVGRINGQFGTVEFVPIHFMHKSIPFEELISLYAV
      TPS_K lactis (283) -KGCKIIIGVDRLDYIKGVPQKLHALEVFLGAHPEWIGKVVLVQVAVPSRGDVEEYQYLRSVVNELVGRINGQFGTAEFVPIHFMHRSIPFQELISLYAV
    TPS_C albicans (270) -KDVKVIVGVDRLDYIKGVPQKLHAFEVFLNENPEWIGKVVLVQVAVPSRGDVEEYQSLRSTVSELVGRINGEFGTVEFVPIHYLHKSIPFDELISLYNI
  TPS_H polymorpha (274) GKDCKLIIGVDRLDYIKGVPQKLHAFEIFLERHPEWIGKVVLIQVAVPSRGDVEEYQSLRAAVNELVGRINGRFGTVEFVPIHFLHKSVNFQELISVYAA
    TPS_P pastoris (268) GQGVKLIVGVDRLDYIKGVPQKLHAFEIFLQEHPEWIGKVVLVQVAVPSRGDVEEYQTLRATVNELVGRINGQFGTIEFVPIHFMHKSVPFEELISLYAV
    TPS_A nidulans (277) -QGVKLMVGVDRLDYIKGVPQKLHALEVFLSDHPEWVGKVVLVQVAVPSRQDVEEYQNLRAVVNELVGRINGKFGTVEFMPIHFLHKSVNFDELIALYAV
     TPS_M grisea  (279) -EGVKLIVGVDRLDYIKGVPQKLHALEVFLTEHPEWIGKIVLVQVAVPSRQDVEEYQNLRAVVNELVGRINGKFGTIEFMPIHFLHQSVSFDELAALYAV
      TPS_S pombe  (284) -QGVKVIVGVDRLDYIKGVPQKFHAFEVFLEQYPEWVGKVVLVQVAVPSRQDVEEYQNLRAVVNELVGRINGRFGTVEYTPIHFLHKSVRFEELVALYNV
  TPS_Y lipolytica (269) -KGIKVVVGVDRLDYIKGVPQKLHAFEVFLSEHPEWIGKVVLVQVAVPSRGLVEEYQNLRAVVNELVGRINGMFGTVEFTPIHFMHRSVDFNELIALYSI
    TPS_A thaliana (359) -AGRKVMLGVDRLDMIKGIPQKILAFEKFLEENANWRDKVVLLQIAVPTRTDVPEYQKLTSQVHEIVGRINGRFGTLTAVPIHHLDRSLDFHALCALYAV
RIP_R solanacearum (324) GR-CVLMIGVDRLDYSKGIPTRLKAFRQLLQTHARMRSKVTLVQIAAPTRQSIPAYARLRDKTEQLVREINRRFGTGDWTPVMYFSESVDRTALPQLYRM
        TPS_E coli (253) -KNVQNIFSVERLDYSKGLPERFLAYEALLEKYPQHHGKIRYTQIAPTSRGDVQAYQDIRHQLENEAGRINGKYGQLGWTPLYYLNQHFDRKLLMKIFRY
TPS_R solanacearum (260) GRPLKLIMSVDRLDYSKGLPERFRAFEQLLDDFPDHRRHVTFVQIAPTSRQDVQSYQQIRQRLEAESGRINGKHSELDWTPIRYINKQYDRRMLMALFRA
TPS_D melanogaster (300) ---MQIILGVDRLDYTKGLVHRLMAFEALLLKYPQHKEKVSLLQISVPSRTDVKEYRELKEEVDQLVGRINGRFTTANWAPIRYIYDYVSQDELAALYRD

                         501                                                                                                600
```

```
   TPS_S cerevisiae  (382) SDVCLVSSTRDGMNLVSYEYIACQ-EEKKGSLILSEFTGAAQSLN-GAIIVNPWNTDDLSDAINEALTLPDVKKEVNWEKLYKYISKYTSAFWGENFVHE
       TPS_K lactis  (382) SDVCLVSSTRDGMNLVSYEYISCQ-EEKKGTLILSEFTGAAQSLN-GALIVNPWNTDDLAESINEALTVPEEKRAANWEKLYKYISKYTSAFWGENFVHE
     TPS_C albicans  (369) SDVCLVSSTRDGMNLVSYEYIACQ-QDRKGVLILSEFAGAAQSLN-GALIVNPWNTEDLSEAIKESLTLPEEKREFNFKKLFTYISKYTSGFWGESFVKE
   TPS_H polymorpha  (374) SDVCVVSSTRDGMNLVSYEYIACQ-QDRKGSLVLSEFAGAAQSLN-GALVVNPWNTEELSEAIYEGLIMSEEKRRGNFQKMFKYIEKYTASYWGENFVKE
     TPS_P pastoris  (368) SDICLVSSTRDGMNLVSYEYIACQ-QQKKGSLILSEFAGAAQSLN-GAIVVNPWNTEELSEAIYEALSLPEEKRESSFESMFKYISKYTAAYWGENFVKE
     TPS_A nidulans  (376) SDACVVSSTRDGMNLVSYEYIATQ-EKRHGSLVLSEFAGAAQSLN-GSIIVNPWNTEELAAYHEAVTMSDEQRALNFSKLDKYVNKYTSAFWGQSFVTE
       TPS_M grisea  (378) SDVCLVSSTRDGMNLVSYEYIATQ-RDRHGVMILSEFTGAAQSLS-GSLIVNPWNTEELANAIHDAVTMGPEQREANFKKLERYVFKYTSAWWGSSFVAE
        TPS_S pombe  (383) SDVCLITSTRDGMNLVSYEYICTQ-QERHGALILSEFAGAAQSLN-GSIVINPWNTEELANSIHDALTMPEKQREANENKLFRYVNKYTSQFWGQSFVGE
   TPS_Y lipolytica  (368) SDVCFVSSTRDGMNLVSYEYVACQ-TEKHGSLILSEFTGAAQSLN-GALIVNPWNTEDMAEALYDSLTFSPEKKAENHRKLFKYVSKYTSQHWGEAFVSE
     TPS_A thaliana  (458) TDVALVTSLRDGMNLVSYEFVACQ-EAKKGVLILSEFAGAAQSLGAGAILVNPWNITEVAASIGQALNMTAEEREKRHRHNFHHVKTHTAQEWAETFVSE
RIP_R solanacearum  (423) SRVGVVTPVADGMNLVAKEYVAAQAARDPGVLVLSRGAGAASQLR-DALLVPSKNRAATADAYAQALSMPLEERKARHAGLMRNIETEDLRWWRDNYLKA
         TPS_E coli  (352) SDVGLVTPLRDGMNLVAKEYVAAQDPANPGVLVLSQFAGAANELT-SALIVNPYDRDEVAAALDRALTMSLAERISRHAEMLDVIVKNDINHWQECFISD
 TPS_R solanacearum  (360) SHIGYVTPLRDGMNLVAKEYVAAQDPEAPGVLVLSRFAGAARELD-AALIVNPYDTRGMAEALNRALTMPLEERKARHAHMMDRLRAADLTAWRERFLAD
 TPS_D melanogaster  (397) AAVCLVTPLRDGMNLVAKEFVACQINEVPGVLVISPFAGAGEMMH-EALLCNPYEVNEAAEVIHRALTMPEDERVLRMARLRRREAECDVSHWMRCFLKA

                           601                                                                                              700
   TPS_S cerevisiae  (480) LYSTSSSSTSSSATKN------------------------------------------------------------------------------------
       TPS_K lactis  (480) LYRLGSSNN-------------------------------------------------------------------------------------------
     TPS_C albicans  (467) LYKCNPQKSLRD----------------------------------------------------------------------------------------
   TPS_H polymorpha  (472) LTRV------------------------------------------------------------------------------------------------
     TPS_P pastoris  (466) LTRPSNISSSVMGQFISQPVTEKFVETYGRPHISMVVGQMQGYRVSMEDAHCCWHKTLEITSFGGFLQKYEIDIVGVFDGHGGKNAAQYVGERLPHIIFG
     TPS_A nidulans  (474) LTRISEQAAGKLPTKETPVNGETSKLETSSQ---------------------------------------------------------------------
       TPS_M grisea  (476) LNRLAAGIEGADAEGSKTKIAIRGVGDAVADGAGKVADAVSSVLPGGDNKTEGN----------------------------------------------
        TPS_S pombe  (481) LQRIQHYSHPHPRRTNPILRTKSAQVLSMNSSS-------------------------------------------------------------------
   TPS_Y lipolytica  (466) LKRC------------------------------------------------------------------------------------------------
     TPS_A thaliana  (557) LNDTVIEAQLRISKVPPELPQHDAIQRYSKSNNRLLILGFNATLTEPVDNQGRRGDQIKEMDLNLHPELKGPLKALCSDPSTTIVVLSGSSRSVLDKNFG
RIP_R solanacearum  (522) LACAPWPAGPEGAEDTRPDAGGASVSGASDGGLTQA----------------------------------------------------------------
         TPS_E coli  (451) LKQIVPRSAESQQRDKVATFPKLA----------------------------------------------------------------------------
 TPS_R solanacearum  (459) LRGASAR---------------------------------------------------------------------------------------------
 TPS_D melanogaster  (496) VGALEMDDVGTTIMQPVSVDDFDDYLLKYIGYNHKLALLLDYDGTLAPIAPHPDLATLS-------PEIKNVLYKLSNHSDVYVAVISGRNVDNVKKMVG

                           701                                                                                              800
   TPS_S cerevisiae  (496) ----------------------------------------------------------------------------------------------------
       TPS_K lactis  (489) ----------------------------------------------------------------------------------------------------
     TPS_C albicans  (479) ----------------------------------------------------------------------------------------------------
   TPS_H polymorpha  (476) ----------------------------------------------------------------------------------------------------
     TPS_P pastoris  (566) LLKEQLVEHKNAIGTRYAETDCLVETINFTHIIKNAFFKCDSELYSRRIKSGSTAVVVIILNGIVFTANIGDSRCILHTGKHDTVKNLSFDHKPKHYGEL
     TPS_A nidulans  (505) ----------------------------------------------------------------------------------------------------
       TPS_M grisea  (530) ----------------------------------------------------------------------------------------------------
        TPS_S pombe  (514) ----------------------------------------------------------------------------------------------------
   TPS_Y lipolytica  (470) ----------------------------------------------------------------------------------------------------
     TPS_A thaliana  (657) -EYDMWLAAENGMFLRLTNGEWMTTMPEHLNMEWVDSVKHVFKYFTERTPRSHFETRDTSLIWNYKYADIEFGRLQARDLLQHLWTGPISNASVDVVQGS
```

```
RIP_R solanacearum  (558) ----------------------------------------------------------------------------------------------------
          TPS_E coli  (475) ----------------------------------------------------------------------------------------------------
TPS_R solanacearum  (466) ----------------------------------------------------------------------------------------------------
TPS_D melanogaster  (589) -IEGITYAGNHGLEILHPDG---SKFVHPMPMEYEKKVSDLLKALQDSVCRDGAWVENKGALLTFHYRETP-N—HLRGAMVDKARSLIEKYGFKATEAH

                          801                                                                                              900
  TPS_S cerevisiae  (496) ----------------------------------------------------------------------------------------------------
        TPS_K lactis  (489) ----------------------------------------------------------------------------------------------------
     TPS_C albicans  (479) ----------------------------------------------------------------------------------------------------
  TPS_H polymorpha  (476) ----------------------------------------------------------------------------------------------------
     TPS_P pastoris  (666) FRIHSDGGHVQANRVGGVLALSRAFGDFTFKCFNQANYARLLNNKKMQARANLEQRNYNNMSVSLEATNKSSLESLTVPEEFQVTAEPDIVIHTITSEDH
     TPS_A nidulans  (505) ----------------------------------------------------------------------------------------------------
       TPS_M grisea  (530) ----------------------------------------------------------------------------------------------------
        TPS_S pombe  (514) ----------------------------------------------------------------------------------------------------
  TPS_Y lipolytica  (470) ----------------------------------------------------------------------------------------------------
     TPS_A thaliana  (756) RSVEVRAVGVTKGAAIDRILGEIVHSKSMTTPIDYVLCIGHFLGKDEDVYTFFEPELPSDMPAIARSRPSSDSGAKSSSGDRRPPSKSTHNNNKSGSKSS
RIP_R solanacearum  (558) ----------------------------------------------------------------------------------------------------
          TPS_E coli  (475) ----------------------------------------------------------------------------------------------------
TPS_R solanacearum  (466) ----------------------------------------------------------------------------------------------------
TPS_D melanogaster  (682) CALEARPPVQWNKGRASIYILRTSFGVDWNERIKIIYVGDDLTDEDAMVALKGMARTFRVTSSDIVKTAADHRLPSTDSVYTLLKWVERHFMGRKARANS

                          901                                                                                   989
  TPS_S cerevisiae  (496) -----------------------------------------------------------------------------------------
        TPS_K lactis  (489) -----------------------------------------------------------------------------------------
     TPS_C albicans  (479) -----------------------------------------------------------------------------------------
  TPS_H polymorpha  (476) -----------------------------------------------------------------------------------------
     TPS_P pastoris  (766) FLILACDGVWDCYNNEPLVRQTRHFLSLDLSLNVVVEKLLD--LCIRKANTITGVGFDNMTLILVALHPLRTVNQWQKKLSSEILAENT
     TPS_A nidulans  (505) -----------------------------------------------------------------------------------------
       TPS_M grisea  (530) -----------------------------------------------------------------------------------------
        TPS_S pombe  (514) -----------------------------------------------------------------------------------------
  TPS_Y lipolytica  (470) -----------------------------------------------------------------------------------------
     TPS_A thaliana  (856) SSSNSNNNNKSSQRSLQSERKSGSNHSLGNSRRPSPEKISWNVLDLKGENYFSCAVGRTRTNARYLLGSPDDVVCFLEKLADTTSSP--
RIP_R solanacearum  (558) -----------------------------------------------------------------------------------------
          TPS_E coli  (475) -----------------------------------------------------------------------------------------
TPS_R solanacearum  (466) -----------------------------------------------------------------------------------------
TPS_D melanogaster  (782) LTYRPTKGDGVQMQMSLEVAASANNLEV-------------------------------------------------------------
```

FIGURE S2: Full amino acid sequence alignment of the Tps1 protein from bacteria, yeasts, filamentous fungi, plant and insect that has been used in this study. This alignment was prepared with the Align X module of the Vector NTI software, which uses the Clustal W algorithm [2], using default parameters and taking *S.cerevisiae* Tps1 as the reference sequence. Perfectly conserved amino acids among the 14 selected sequences appear as red font on grey background. The U and G letters in the top line of the alignment depict the amino acids that have been attributed to UDP-glcand G6P binding, respectively [1, 3].

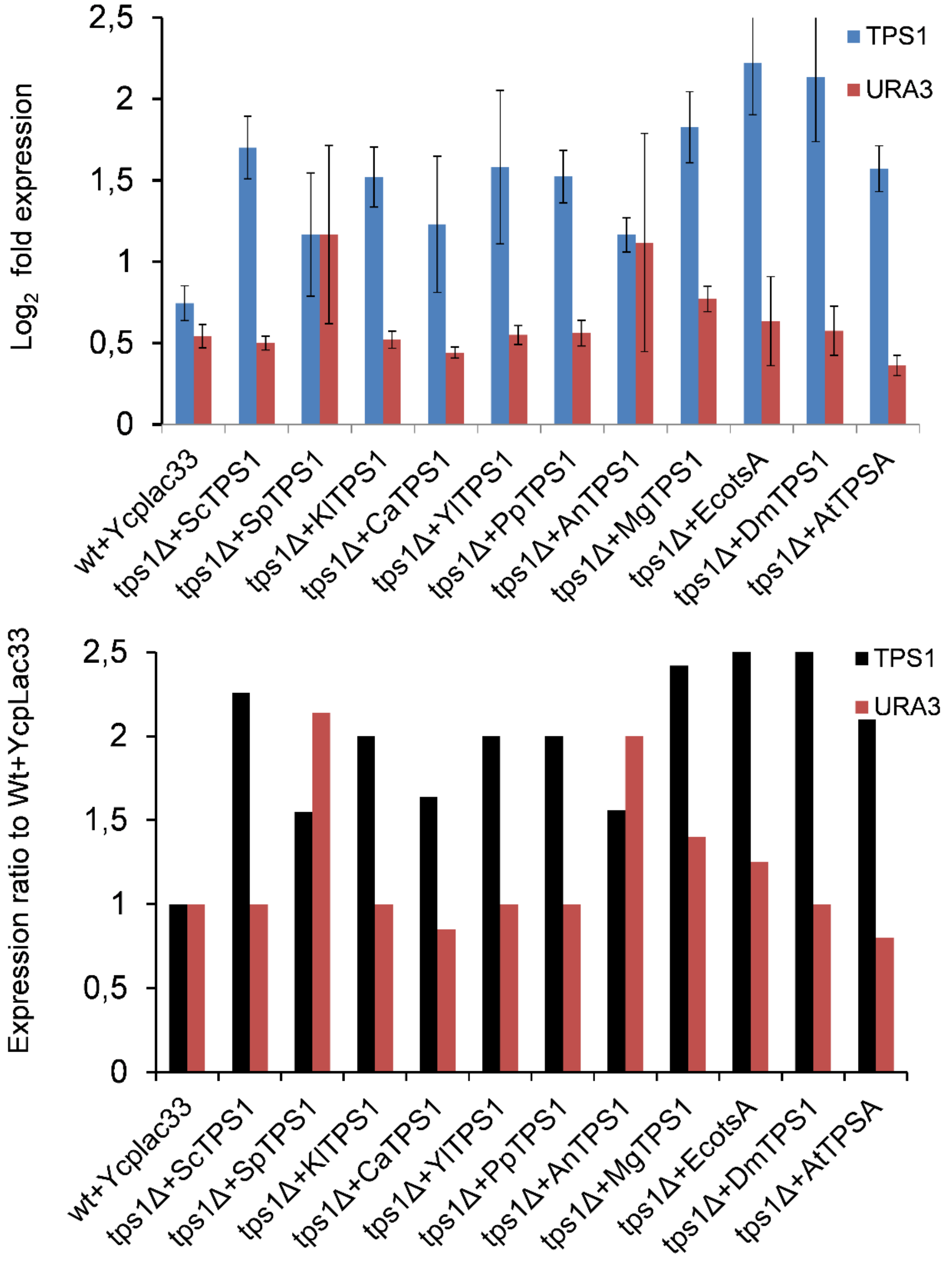

FIGURE S3: Expression level of *TPS1* and *URA3* in *Sctps1*Δ mutant transformed with *TPS1* from different organisms. The *Sctps1*Δ strain transformed with YcpLac33 plasmid bearing *TPS1* homologues was cultivated on YN glucose 2% till OD_{600} 5 units. Samples (15 OD units) were taken to determine the transcript levels of *TPS1* and *URA3* by qRT-PCR. *TAF10* and *KRE11* genes were used as controls for normalization following the recommendation of Teste *et al* [4]. In A) are shown the absolute changed normalized to reference genes and B) is shown the mean expression level of *TPS1* homologous gene and *URA3* measured in the Sc*tps1*Δ transformed with plasmid bearing these genes with respect to the mean expression level of the same transcripts in the wild type (WT) bearing the *TPS1* in the genome and *URA3* on the YcpLac33.

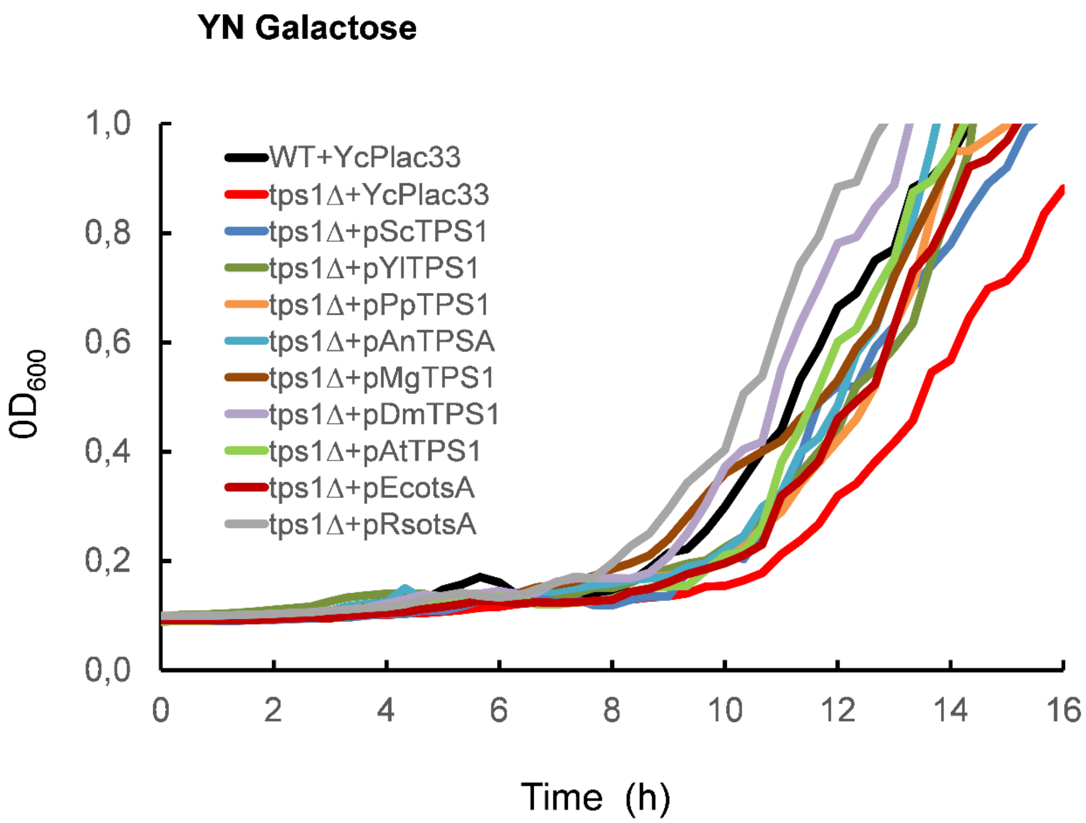

FIGURE S4: Growth kinetic of *S. cerevisiae tps1Δ* mutant transformed with *TPS1* homologues of different organisms on YN galactose (A) and YN ethanol (B) in microtiter plate. The *Sctps1Δ* strain transformed with YCpL33 plasmid bearing *TPS1* homologue was cultivated on YN Trehalose 1% till OD_{600} 5 unit. Then, the yeast cells were inoculated in 250 µL of YN containing galactose 2% (w/v) or ethanol 2% (v/v) at initial OD_{600} of 0.1 unit.

A : trehalose versus Tps1 activity

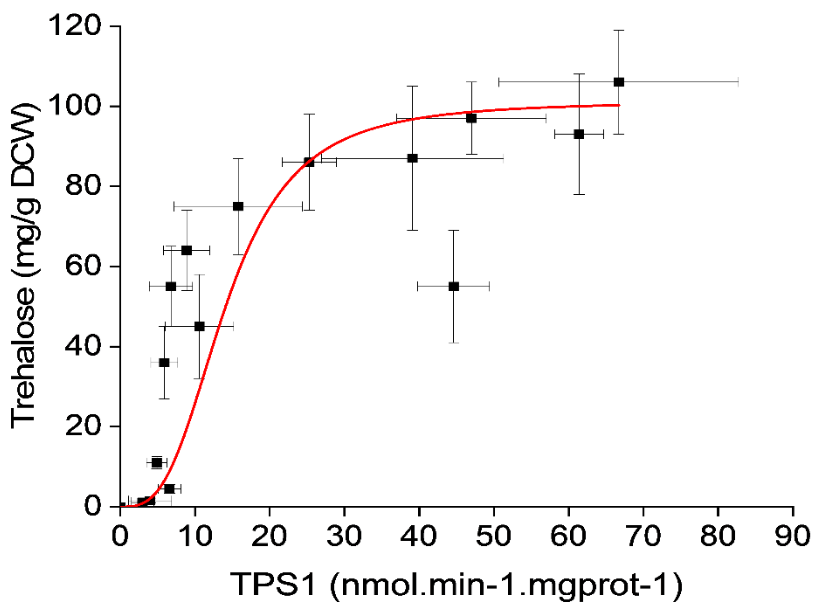

B : growth rate versus T6P

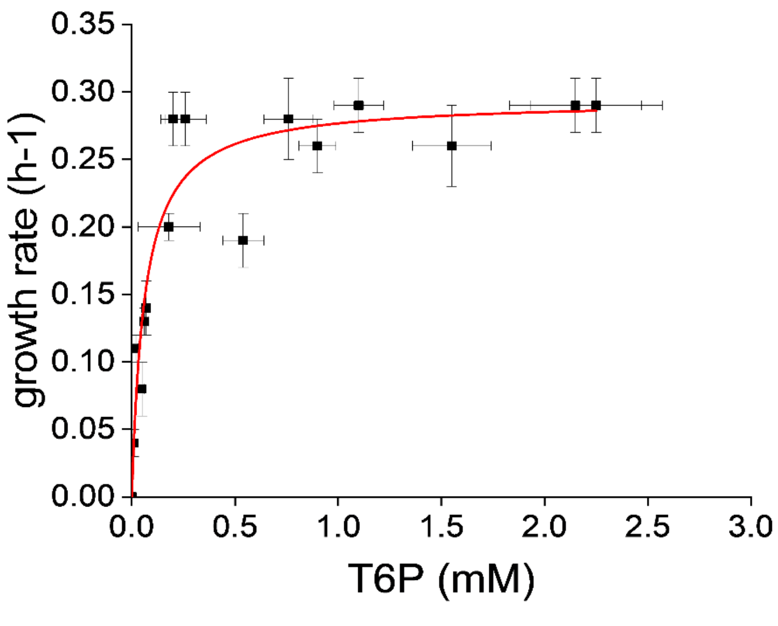

FIGURE S5: **Relationship between Tps1 activity and trehalose levels (A) and T6P concentration, and growth rate (B) in *Sctps1Δ* mutant expressing *TPS1* homologous gene from various origins.** The growth rate on glucose, Tps1 activities and trehalose levels are taken from Table 1 and 2. Origin 2018 software (OriginLab, https://www.originlab.com/) was used to fit the data using the non-iterative fitting algorithm (Levenberg-Marquardt), selecting with the Michaelis-Menten or Hill models in the software.

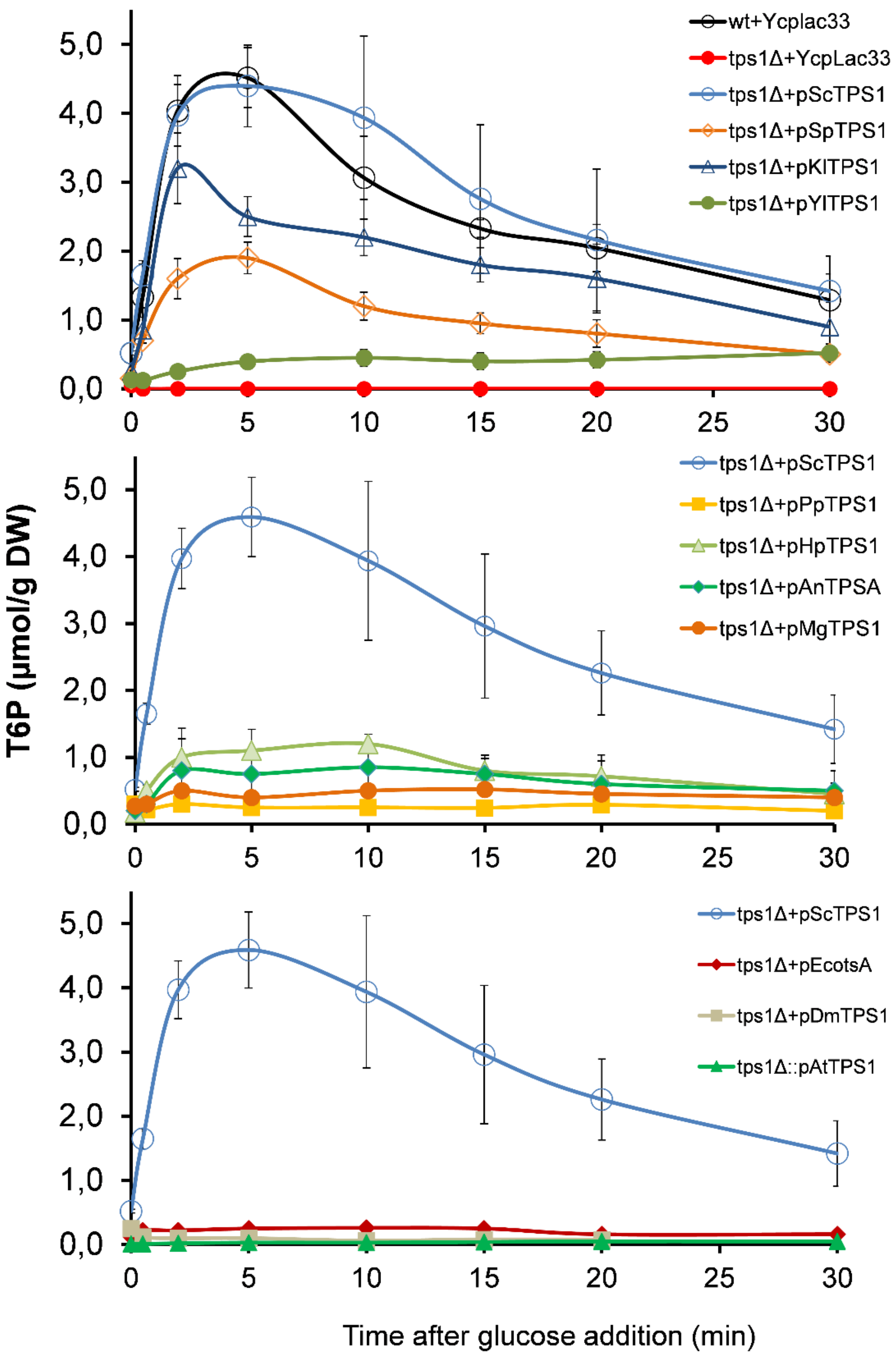

FIGURE S6: Dynamic of T6P concentration in response of *S. cerevisiae tps1*Δ mutant expressing *TPS1* from different organisms to a glucose pulse. The *Sctps1*Δ strain transformed with YcpLac33 plasmid bearing *TPS1* homologue was cultivated on YN Trehalose 1% till OD_{600} 5 unit. Then, glucose (10 g/L final concentration) was added and samples (5 ml) at different times were rapidly collected by filtration for intracellular metabolite extraction and quantification. In **(A)** are shown results with Sc*tps1*Δ complemented with *S. cerevisiae TPS1* and transformed with *S. pombe*, *K.lactis*, and *C albicans TPS1* homologues. In **(B)** are shown results with Sc*tps1*Δ transformed with *P. pastoris, Y lipolytica, H.polymorpha, A. nidulans* and *M. grisea TPS1* homolog. In **(C)** are shown results with Sc*tps1*Δ transformed with *D.melanogaster and A. thaliana, E. coli, R. solanacearum otsA* and *R. solanaceraum rip1* homologous gene. Results shown are the mean of three independent experiments.

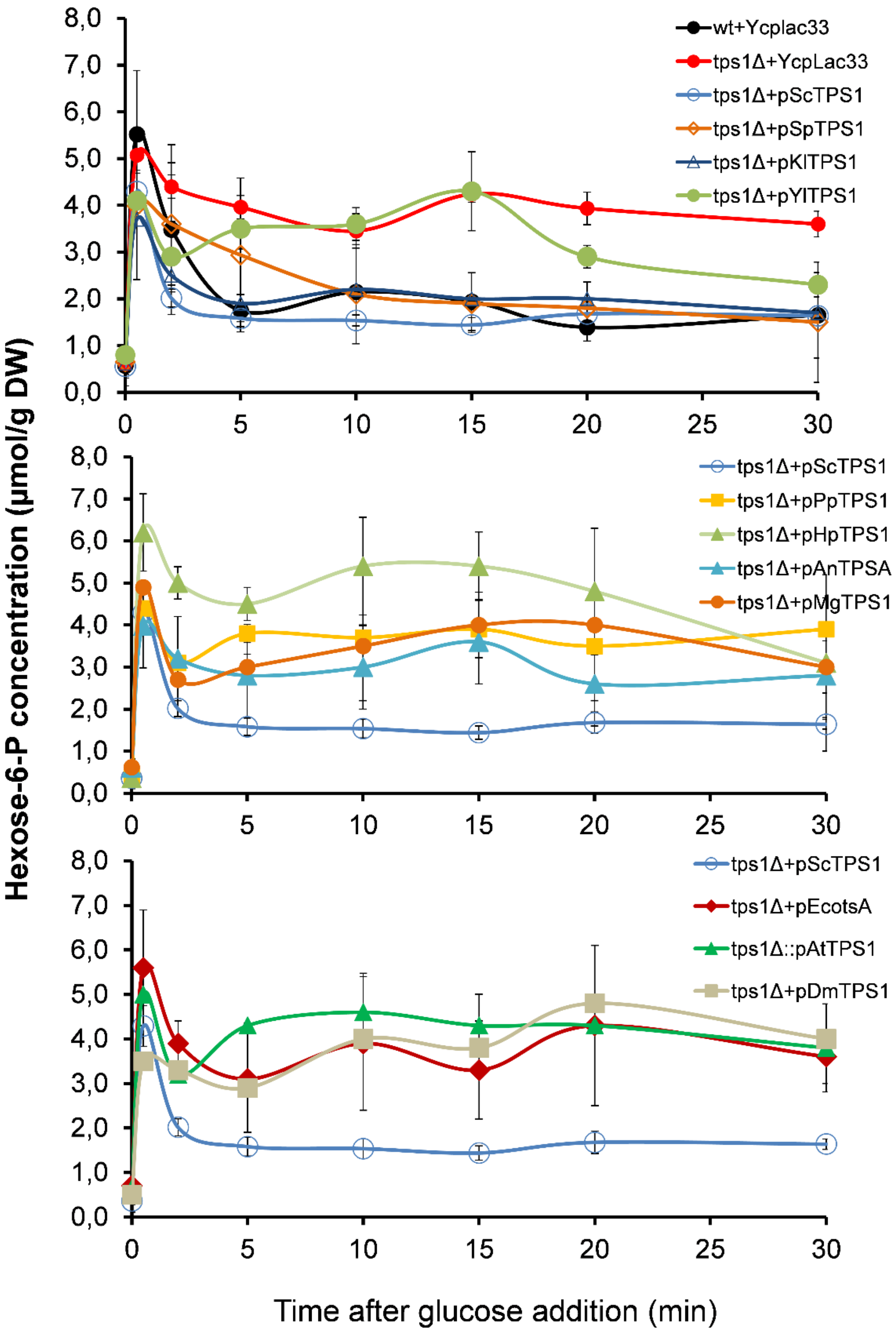

FIGURE S7: Dynamic of hexose-6-phosphate (H6P) concentration in response of *S. cerevisiae tps1Δ* mutant expressing *TPS1* from different organisms to a glucose pulse. Same as in Figure S6.

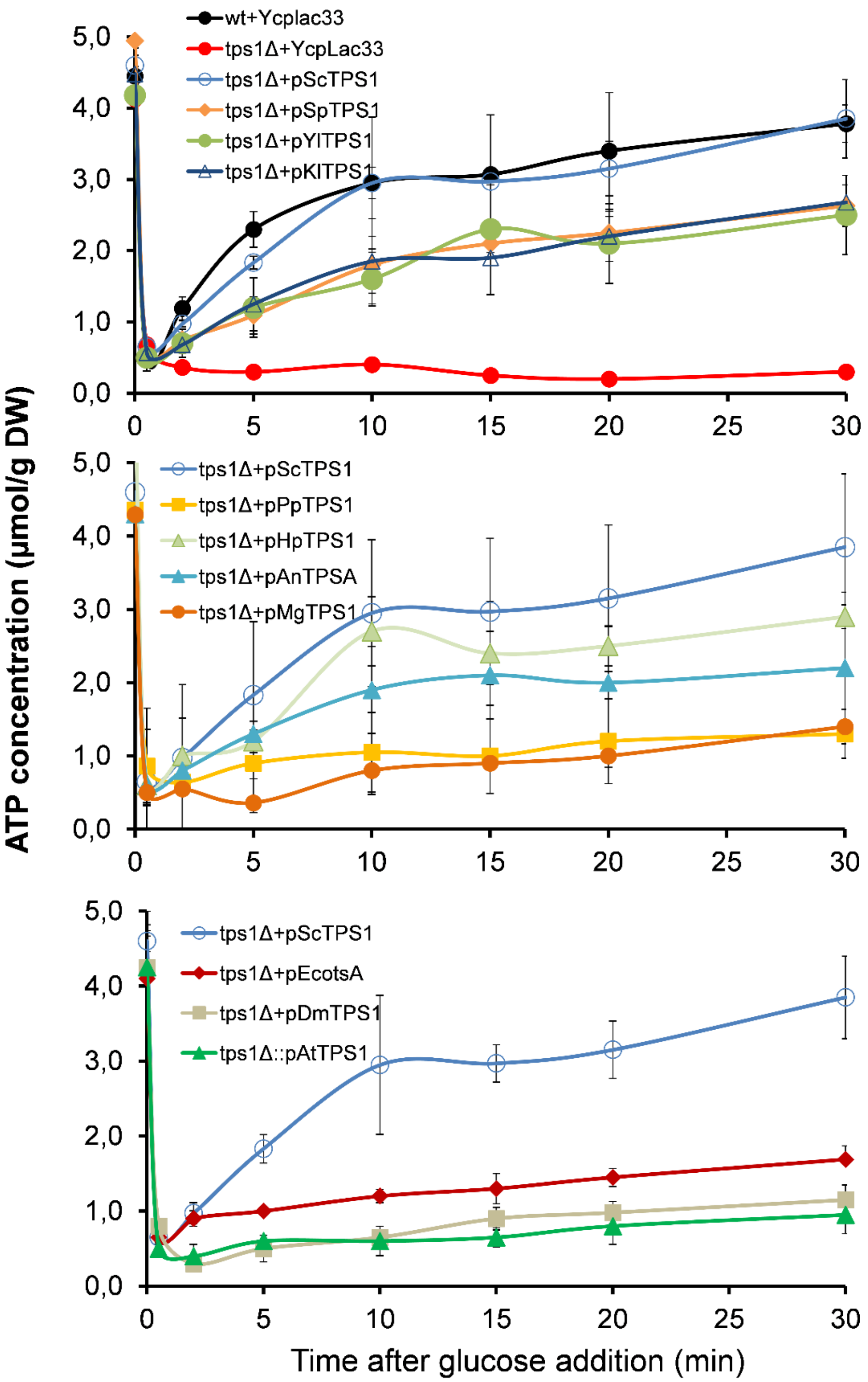

FIGURE S8: **Dynamic of ATP concentration in response of *S. cerevisiae tps1*Δ mutant expressing *TPS1* homologues from different organisms to a glucose pulse.** Same as in Figure S6.

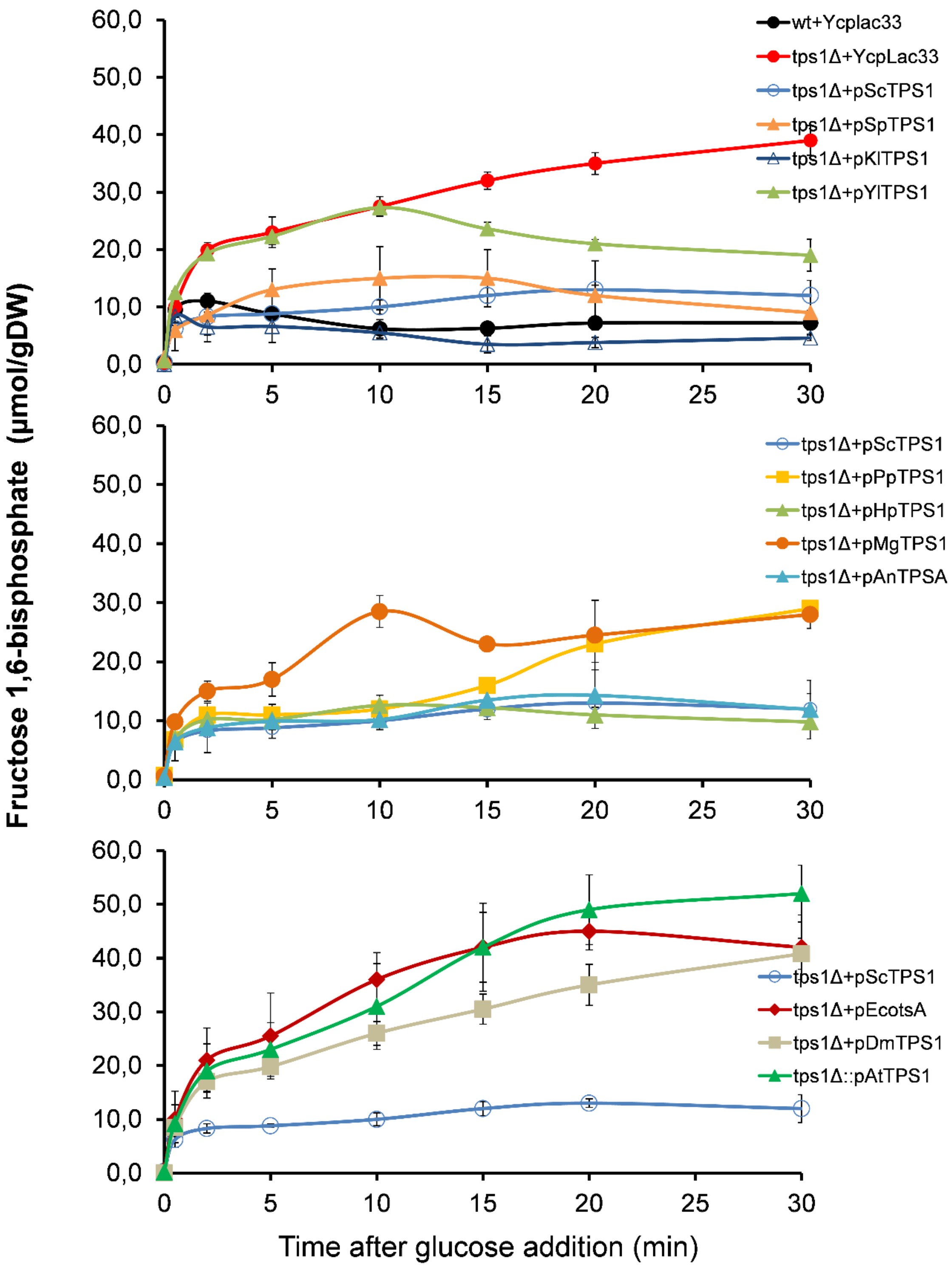

FIGURE S9: **Dynamic of fructose 1,6 bisphosphate (FBP) concentration, in response of *S. cerevisiae tps1*Δ mutant expressing *TPS1* homologue from different organisms to a glucose pulse.** Same as in Figure S6.

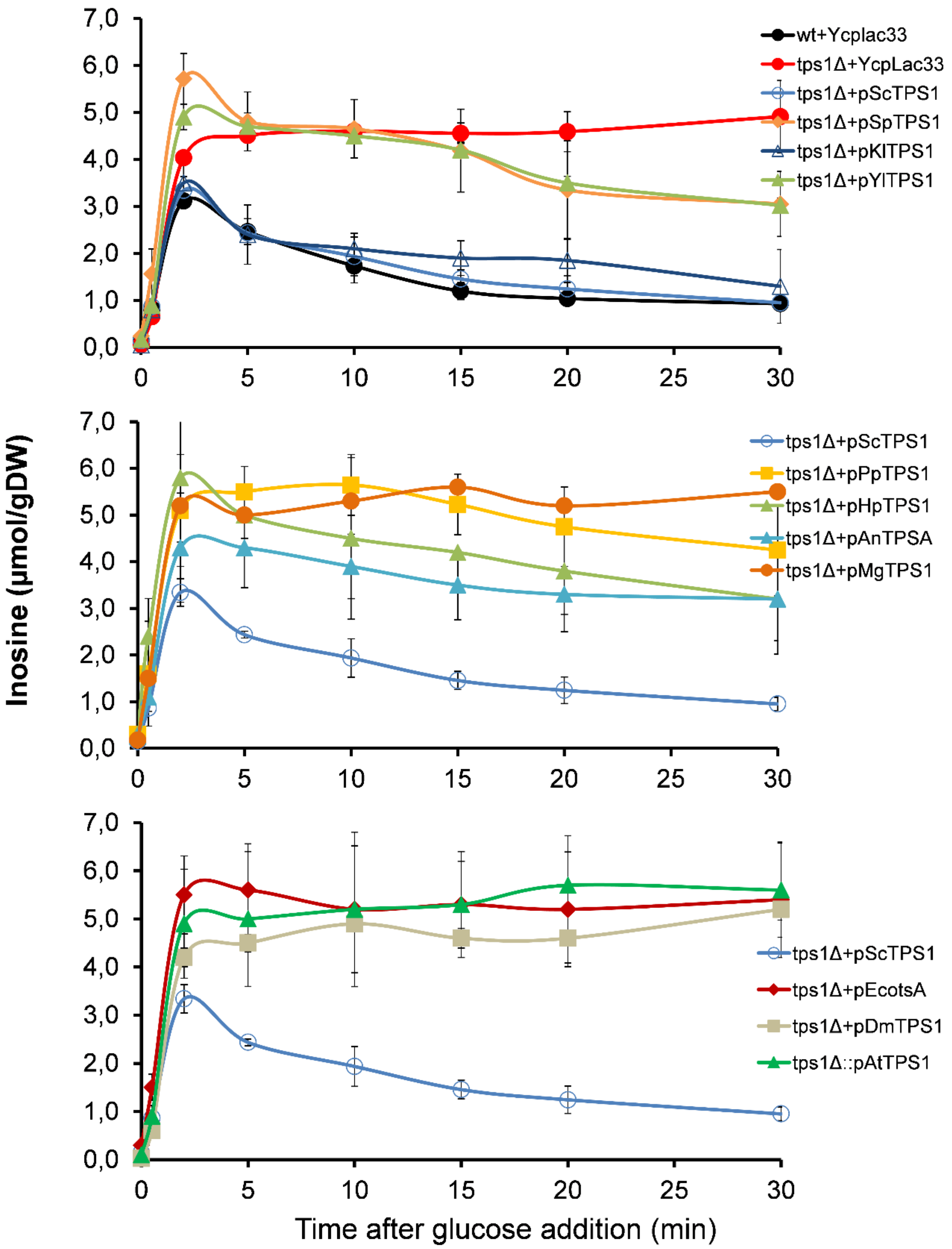

FIGURE S10: **Dynamic of inosine concentration in response of *S. cerevisiae tps1*Δ mutant expressing *TPS1* homologue from different organisms to a glucose pulse. Same procedure as in Figure S6, except that levels of inosine are shown.** Results are the mean of the three independent experiments.

REFERENCES

1. Gibson RP, Turkenburg JP, Charnock SJ, Lloyd R, Davies GJ (**2002**). Insights into trehalose synthesis provided by the structure of the retaining glucosyltransferase OtsA. **Chem Biol** 9(12): 1337-1346. doi: 10.1016/s1074-5521(02)00292-2

2. Thompson JD, Higgins DG, Gibson TJ (**1994**). CLUSTAL W: improving the sensitivity of progressive multiple sequence alignment through sequence weighting, position-specific gap penalties and weight matrix choice. **Nucleic Acids Res** 22(22): 4673-4680. doi: 10.1093/nar/22.22.4673

3. Gibson RP, Tarling CA, Roberts S, Withers SG, Davies GJ (**2004**). The donor subsite of trehalose-6-phosphate synthase: binary complexes with UDP-glucose and UDP-2-deoxy-2-fluoro-glucose at 2 A resolution. **J Biol Chem** 279(3): 1950-1955. doi: 10.1074/jbc.M307643200

4. Teste MA, Duquenne M, Francois JM, Parrou JL (**2009**). Validation of reference genes for quantitative expression analysis by real-time RT-PCR in *Saccharomyces cerevisiae*. **BMC Mol Biol** 10:99. doi: 10.1186/1471-2199-10-99
